# (Dis‐)Connected Dots in Dementia with Lewy Bodies—A Systematic Review of Connectivity Studies

**DOI:** 10.1002/mds.29248

**Published:** 2022-10-17

**Authors:** Annegret Habich, Lars‐Olof Wahlund, Eric Westman, Thomas Dierks, Daniel Ferreira

**Affiliations:** ^1^ Division of Clinical Geriatrics, Center for Alzheimer Research, Department of Neurobiology, Care Sciences and Society Karolinska Institutet Stockholm Sweden; ^2^ University Hospital of Psychiatry and Psychotherapy University of Bern Bern Switzerland; ^3^ Department of Neuroimaging, Centre for Neuroimaging Sciences Institute of Psychiatry, Psychology and Neuroscience, King's College London London UK

**Keywords:** dementia with Lewy bodies, connectivity, network, biomarkers, neuroimaging

## Abstract

Studies on dementia with Lewy bodies (DLB) have mainly focused on the degeneration of distinct cortical and subcortical regions related to the deposition of Lewy bodies. In view of the proposed trans‐synaptic spread of the α‐synuclein pathology, investigating the disease only in this segregated fashion would be detrimental to our understanding of its progression. In this systematic review, we summarize findings on structural and functional brain connectivity in DLB, as connectivity measures may offer better insights on how the brain is affected by the spread of the pathology. Following Preferred Reporting Items for Systematic Reviews and Meta‐Analyses (PRISMA) guidelines, we searched Web of Science, PubMed, and SCOPUS for relevant articles published up to November 1, 2021. Of 1215 identified records, we selected and systematically reviewed 53 articles that compared connectivity features between patients with DLB and healthy controls. Structural and functional magnetic resonance imaging, positron emission tomography, single‐positron emission computer tomography, and electroencephalography assessments of patients revealed widespread abnormalities within and across brain networks in DLB. Frontoparietal, default mode, and visual networks and their connections to other brain regions featured the most consistent disruptions, which were also associated with core clinical features and cognitive impairments. Furthermore, graph theoretical measures revealed disease‐related decreases in local and global network efficiency. This systematic review shows that structural and functional connectivity characteristics in DLB may be particularly valuable at early stages, before overt brain atrophy can be observed. This knowledge may help improve the diagnosis and prognosis in DLB as well as pinpoint targets for future disease‐modifying treatments. © 2022 The Authors. *Movement Disorders* published by Wiley Periodicals LLC on behalf of International Parkinson and Movement Disorder Society.

## Graphical abstract

Dementia with Lewy bodies (DLB) is characterized by widespread connectivity disruptions compared to healthy elderly controls. Across the reviewed structural and functional neuroimaging modalities disruptions emerge most consistently in frontoparietal, default mode, and visual networks. Impairments in brain connectivity measures were associated with clinical features of DLB patients.

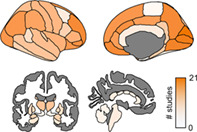

Dementia with Lewy bodies (DLB) is a common reason for a dementia diagnosis in old age, being the primary diagnosis of approximately 5% of patients with dementia.^1^ Clinically, patients with DLB present with visual hallucinations (VHs), cognitive fluctuations, parkinsonism, and rapid eye movement sleep disorders.^2^ Neuropathologically, the hallmarks of DLB are the eponymous Lewy bodies, which consist of abnormal aggregates of α‐synuclein. In the absence of an available biomarker to assess the α‐synuclein pathology directly, the most recent guidelines recommend a set of neuroimaging biomarkers to aid diagnosis.^2^ These biomarkers include preservation of medial temporal lobe structures in magnetic resonance imaging (MRI), presence of the so‐called cingulate island sign on fluorodeoxyglucose (FDG) positron emission tomography (PET) scans, reduced uptake of the dopamine transporter in single‐photon emission tomography (SPECT) or PET, and high relative powers in pre‐alpha/theta frequency bands in electroencephalography (EEG). Although these indirect manifestations of DLB can improve diagnosis, their restriction to specific brain areas neglects an important aspect of α‐synuclein pathology—its spread during disease progression. Starting from the dorsal nucleus of the vagus nerve and the olfactory bulb at the early stages of DLB, an increasing number of cortical and subcortical regions accumulates Lewy bodies at later stages of the disease.^3^, ^4^, ^5^ Animal research demonstrated that Lewy bodies propagate via axonal transport and transsynaptic pathways to areas connected to the initial site, where misfolded protein aggregates induce the same structural alterations in previously normal α‐synuclein in a prion‐like manner.^6^

Neural connections ensure global communication and functional integration of information between brain regions. However, they also provide the scaffold for the transneuronal spread of α‐synuclein aggregates. Thus, investigating neural connectivity and brain network features in DLB may improve our understanding of the disease. These features may not only facilitate tracking of the disease progress but also may help pinpoint potentially “moving” treatment targets adjusted for the stage of the disease.

Moreover, disruptions of the brain connectome, rather than atrophy or hypometabolism of a specific region, may produce the observed behavioral impairments in DLB.^7^

This systematic review aims to provide a comprehensive overview of connectivity studies in DLB, spanning a variety of in vivo neuroimaging methods. We cover the integrity and disruption of specific connections as well as evaluate the impact of DLB on the global network efficiency. Furthermore, we outline the relationship between connectivity measures and the clinical phenotype of DLB. Finally, we discuss the potential of connectivity measures to inform clinical procedures in DLB.

## 1 Methods

### 1.1 Data Source

Following the Preferred Reporting Items for Systematic Reviews and Meta‐Analyses (PRISMA) guidelines,^8^ we conducted a systematic search in the Web of Science, PubMed, and SCOPUS databases on studies published prior to November 1, 2021. The following search terms (S1 in Appendix S1) were applied to the title and abstract: (“dementia with Lewy bodies” OR “Lewy body dementia”) AND (“connecti*” OR “network” OR “graph*”). No restriction was applied on neuroimaging methods to capture the entire spectrum of connectivity measures. The reference lists of all included articles as well as relevant reviews were manually searched to identify additional studies.

### 1.2 Selection Criteria

**FIG 1:**
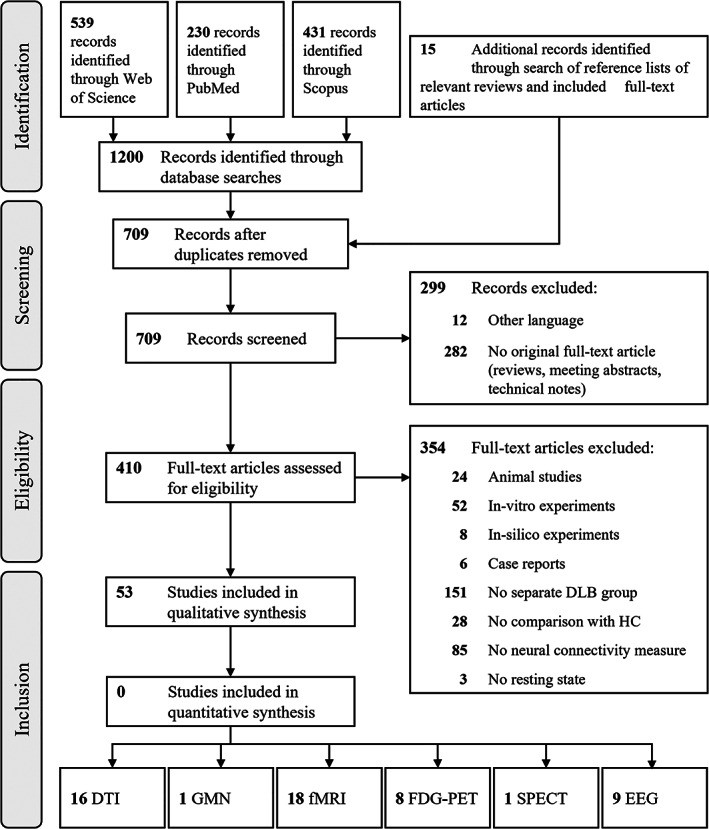
Preferred Reporting Items for Systematic Reviews and Meta‐Analyses (PRISMA) flowchart of study selection. DLB, dementia with Lewy bodies; DTI, diffusion tensor imaging; EEG, electroencephalography; FDG, fluorodeoxyglucose; fMRI, functional magnetic resonance imaging; GMN, gray matter network; HC, healthy control; PET, positron emission tomography; SPECT; single‐positron emission computer tomography.

First, we screened abstracts and selected original articles written in English (Fig. 1). Subsequently, full‐text articles were assessed for eligibility and included if they reported (1) in vivo (2) neural connectivity measures (3) at rest in (4) patients with DLB (5) compared with elderly healthy controls (HCs). Studies conducted in animals as well as case reports were excluded. Publications from the same research group were closely examined for overlapping data sets (S2 in Appendix S1).

The selection process was primarily conducted by a single researcher (A.H.) and spot checked by a second researcher (D.F.). Uncertainties regarding the inclusion of articles were resolved in discussions among the two researchers.

### 1.3 Assessment of Bias and Quality

The quality of all included studies was assessed using the 10‐item checklist for case‐control studies developed by the Joanna Briggs Institute (JBI) Collaboration.^9^ Therein, scores of 9 to 10 were considered high, 7 to 8 moderate, and ≤6 low quality.

## 2 Results

### 2.1 Study Characteristics

**FIG 2:**
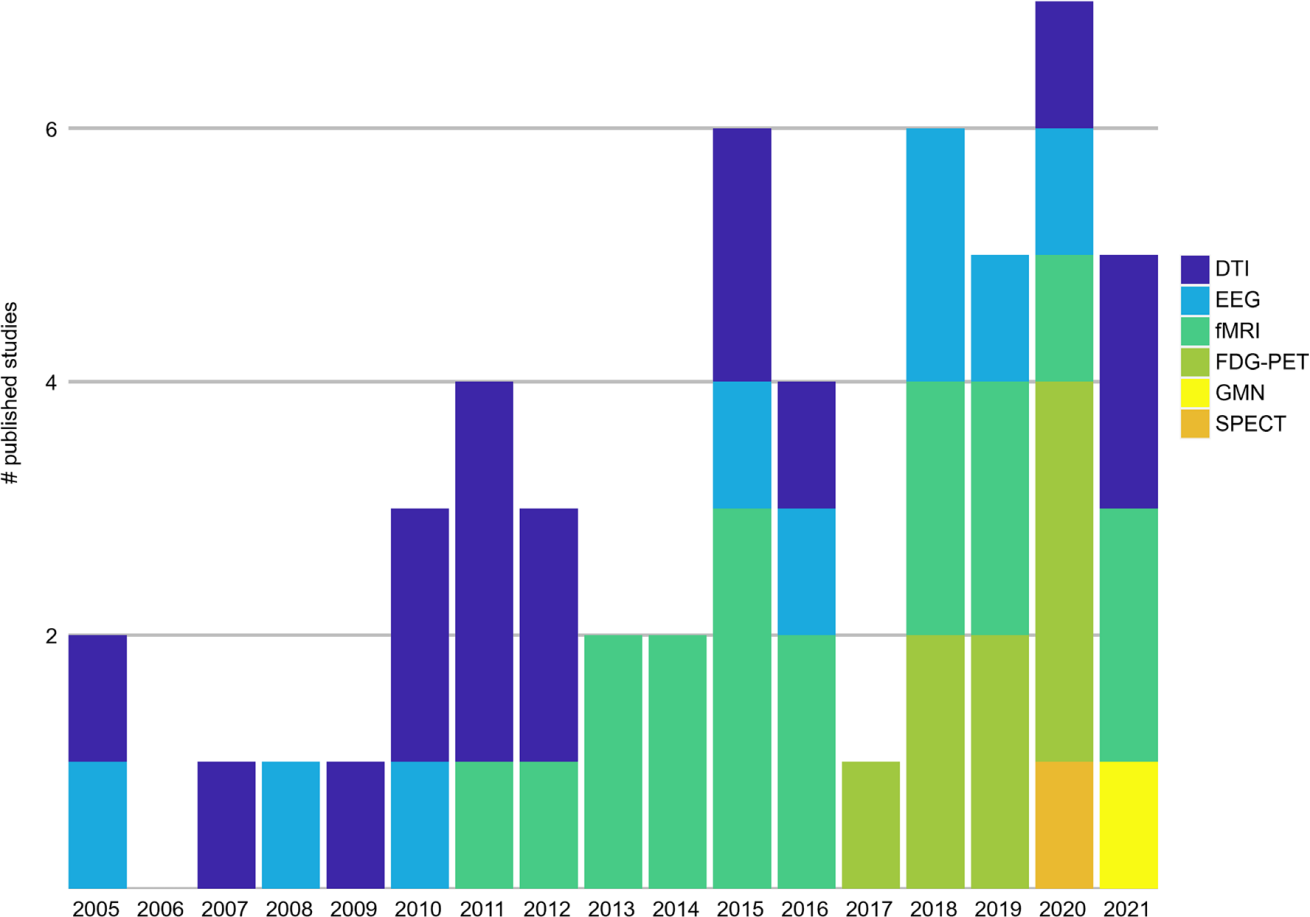
Published connectivity studies in dementia with Lewy bodies by year and imaging modality. DTI, diffusion tensor imaging; EEG, electroencephalography; FDG, fluorodeoxyglucose; fMRI, functional magnetic resonance imaging; GMN, gray matter network; PET, positron emission tomography; SPECT, single‐positron emission computer tomography. [Color figure can be viewed at wileyonlinelibrary.com]

In our search, we identified a total of 1215 records, of which 53 fit our inclusion criteria (Fig. 1). Thereof, 16 studies based their connectivity analyses on diffusion tensor imaging (DTI), eight studies on FDG‐PET, 17 studies on functional MRI, eight studies on EEG, and one study each on gray matter networks (GMNs) and SPECT. Of note, earlier connectivity studies mainly relied on DTI and EEG, while the use of other imaging modalities was established more recently (Fig. 2).

According to JBI criteria, 73.6% of the included studies were of moderate and high quality and 26.4% were of low quality (S3 in Appendix S1). Points were most frequently deducted for insufficient matching of DLB and HC groups, incomplete identification of confounders, and incomplete descriptions of imaging methods.

Because these points concern reporting standards and all studies complied with the methodological standards of the respective imaging field, all results can be considered reliable.

Cohort characteristics are summarized in Table 1. Across studies, cohorts occasionally (partially) overlapped (S2 in Appendix S1). Results of all studies were included in the qualitative overview. If the same cohort was evaluated with similar methods, this overlap is indicated in the text. All studies except four^10^, ^11^, ^12^, ^13^ specified that patients were diagnosed according to standardized clinical diagnostic criteria for DLB^2^, ^14^, ^15^ or prodromal DLB.^16^

**TABLE 1:** Sample characteristics

Demographic variables	DLB	HCs
Number of subjects per study, mean ± SD, range	27.1 ± 17.2, 10–84	32.9 ± 25.7, 10–142
Percentage of females per study, mean ± SD^a^	35.0 ± 17.5	43.2 ± 19.1
Age, years, mean ± SD^a^	74.7 ± 3.3	72.2 ± 4.2
MMSE, mean ± SD^a^	21.2 ± 2.6	28.8 ± 0.4

^a^ Sex distribution, age, and MMSE were not reported in all studies.

Abbreviations: DLB, dementia with Lewy bodies; HCs, healthy controls; SD, standard deviation; MMSE, Mini Mental State Examination.

### 2.2 Diffusion Tensor Imaging

Among all modalities covered by this review, DTI provides the most straightforward assessment of structural neural connections. DTI measures the displacement of water molecules as restricted by tissue boundaries.^17^ Tissue disruptions are indicated by increased mean diffusivity and decreased fractional anisotropy. DTI allows the assessment of global, regional, and tract‐specific features, which is reflected by the organization of the following section.

**FIG 3:**
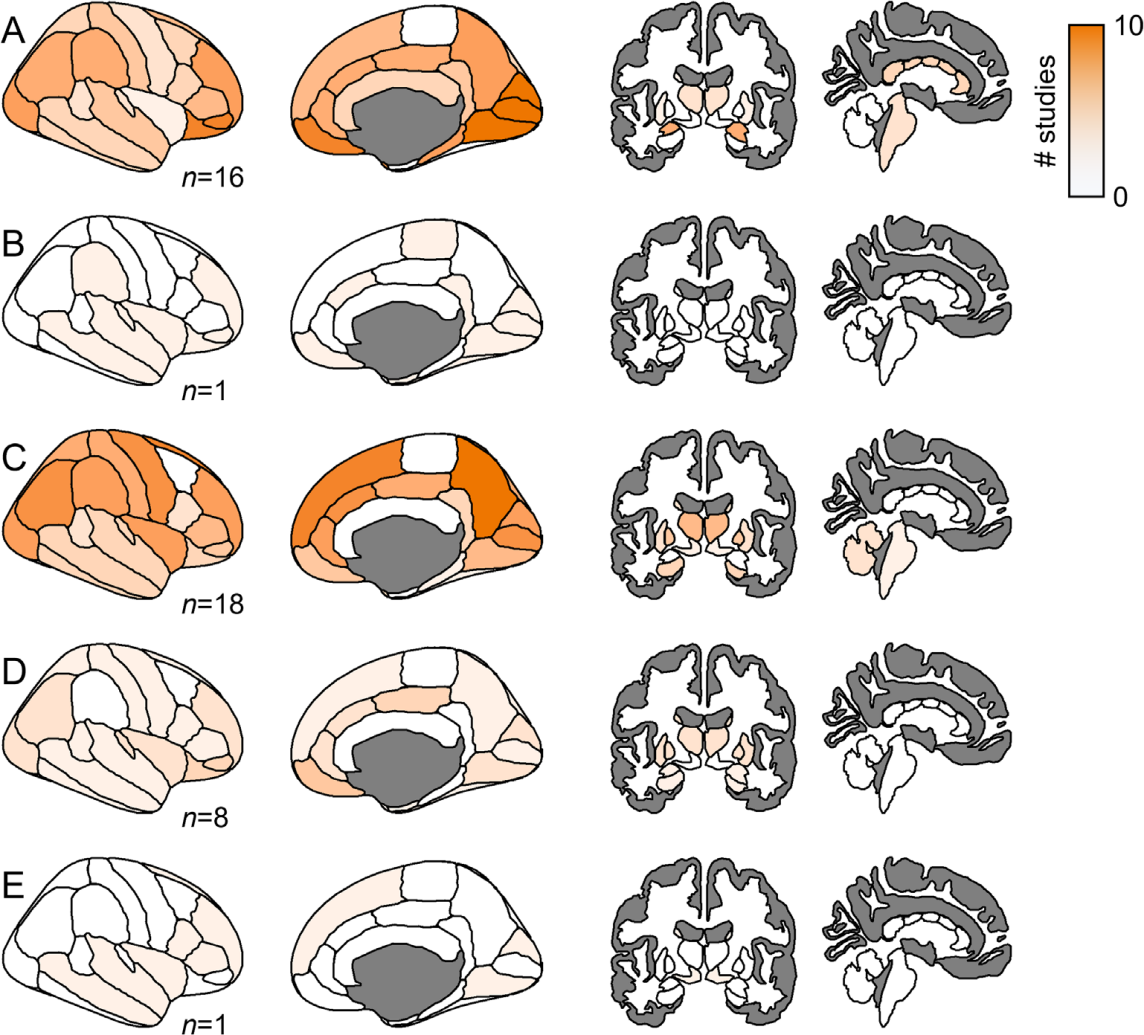
Brain regions showing significant differences in connectivity in patients with dementia with Lewy bodies compared with healthy controls in (**A**) diffusion tensor imaging, (**B**) gray matter networks, (**C**) functional magnetic resonance imaging, (**D**) fluorodeoxyglucose positron emission tomography, and (**E**) single‐positron emission computer tomography. Note that the color intensity should be interpreted in relation to the number of studies per imaging modality. Findings from electroencephalography studies were not included in this figure because of the low spatial resolution of this technique. For white matter tracts in diffusion tensor imaging, differences were mapped for the start and end points of the respective tracts. [Color figure can be viewed at wileyonlinelibrary.com]

In DLB, DTI studies have indicated widespread disruptions of white matter (WM) tracts; their exact location, however, varies between reports possibly because of the variable choices and definitions of regions of interest (ROIs) (S4 in Appendix S1; Fig. 3A). In the absence of WM volume reductions in patients with DLB compared with HCs, Bozzali and colleagues found WM disruptions in areas with long connecting fibers.^18^ Prominent disruptions occurred in the frontal, parietal, occipital, callosal, and pericallosal areas. In another study, WM disruptions in patients with DLB emerged in frontal, temporal, insular, cingular, and visual association areas.^19^ In contrast, in two studies, WM disruptions were largely confined to the parietal and occipital regions with a relative sparing of the frontal regions.^20^, ^21^ In the only study to date to investigate longitudinal changes in WM structure in DLB, Firbank and colleagues^22^ followed up on the participants of the study by Watson and colleagues.^20^ After 1 year, no additional WM changes emerged in patients with DLB, prompting the authors to suggest that disruptions of WM tracts may be an early marker in DLB, which undergoes only minor progression over time.

In a cross‐sectional study by Firbank and colleagues,^23^ patients with DLB displayed WM disruptions exclusively in the precuneus. In contrast, Kantarci and colleagues did not find any differences in WM tracts in the precuneus of patients with DLB when compared with HCs.^24^ Instead, only the inferior longitudinal fasciculus (ILF) of patients with DLB exhibited WM disruptions.

With the featured disruption in the ILF, the study conducted by Kantarci and colleagues lines up with eight other studies that provided insights into disruptions occurring in specific WM tracts in DLB. Although some of these studies selected a priori visual association fibers^25^ or fibers in the language network,^26^ in other studies, the importance of these tracts arose from a whole‐brain approach.^27^ Across studies, WM disruptions manifested most consistently in the ILF^25^, ^27^, ^28^, ^29^, ^30^ as well as in the uncinate fasciculus^26^, ^27^, ^28^, ^29^, ^30^ (but compare to Firbank and colleagues^31^). The superior longitudinal fasciculus^27^, ^30^, ^32^ and the inferior fronto‐occipital fasciculus^26^, ^27^, ^30^ have also been repeatedly shown to be disrupted in patients with DLB.

In addition, the cingulum was disrupted in patients with DLB compared with HCs.^27^, ^29^, ^32^ In contrast, Schumacher and colleagues did not find WM disruptions in the cingulum.^33^ Beyond the disruptions seen in association fibers, disruptions manifested also in the corpus callosum of patients with DLB.^27^, ^32^

Following up on previous evidence of cholinergic deficits and volume reductions in the nucleus basalis of Meynert (NBM) in DLB, Schumacher and colleagues performed tractography on NBM pathways.^33^ Patients with DLB presented with WM disruptions in the lateral and medial NBM pathways. However, the observed disruption in the medial NBM pathway lost significance when controlling for mean diffusivity in whole‐brain WM and may thus not represent a tract‐specific feature but instead a more global disruption of WM in DLB.

### 2.3 Functional Magnetic Resonance Imaging

In the context of fMRI, connections between brain regions are established based on their covariation in the blood‐oxygen‐level‐dependent (BOLD) signal over time. For resting‐state functional connectivity, BOLD activity is not prompted by a specific task but reflects spontaneous fluctuations, which are thought to reflect neural baseline activity. In accordance with the disruptions observed in structural networks in patients with DLB, functional networks also experience local and global disruptions (S6 in Appendix S1; Fig. 3C).

With regard to local connectivity, Borroni and colleagues showed a reduction in local coherence in posterior brain regions.^34^ A study by Schumacher and colleagues, on the other hand, reported reductions in within‐network connectivity restricted to the motor, temporal, and frontal areas.^35^ These divergent findings may be attributable to the assessment of slightly different spatial scales. A predominance of local disruption in the posterior and temporal regions of patients with DLB received support by an fMRI study that conducted graph‐theoretical analyses that revealed a decrease in nodal degree and betweenness centrality in the parietal and temporal cortices.^36^ Apart from local disruptions, this study also demonstrated that desynchronization between brain regions predominantly affected middle‐ and long‐range connections across the entire brain in DLB.^36^

Apart from these more explorative approaches, most fMRI‐based connectivity articles focused on specific resting‐state networks. Several studies found a reduction of functional connectivity within the frontoparietal network (FPN)^37^, ^38^ as well as between the FPN and default mode network (DMN)^39^ and dorsal attention network (DAN^40^). In contrast, Chabran and colleagues showed an increase in connectivity between a left temporal FPN ROI and a left visuoperceptual ROI, whereas the correlation between visuoperceptual ROIs was decreased.^39^ In combination with a study by Sourty and colleagues,^41^ these findings point to a hemispheric asymmetry of functional connectivity differences in patients with DLB compared with HCs.

Regarding the DMN of patients with DLB, many studies revealed a decrease in within‐network connectivity as well as disrupted connections between DMN regions and other brain regions. In a study by Galvin and colleagues, the bilateral precuneus of patients with DLB exhibited increased connectivity to the putamen and parietal regions and decreased connectivity to the frontal and visual areas as well as the hippocampus.^42^ Indeed, disruptions involving DMN regions (especially the frontal and parietal areas) of patients with DLB are a recurring finding.^38^, ^39^, ^43^, ^44^ However, other studies observed aberrant connectivity of DMN regions in the opposite direction, possibly because of different spatial assumptions underlying the analytic procedures.^40^, ^45^, ^46^ Two of these studies, which were conducted in the same cohort, revealed elevated connectivity from the posterior cingulate cortex to the anterior cingulate cortex, globus pallidus, and anterior and posterior lobes as well as increased connections from DMN subregions to subcortical areas.^45^, ^46^ Furthermore, Chabran and colleagues demonstrated an increased coupling of the DMN with both the salience network and FPN, which they interpreted as a compensatory process to counteract disruptions within and between attention networks.^40^ However, other studies did not find any alterations in the DMN in DLB compared with HCs.^37^, ^47^, ^48^ Despite the absence of functional connectivity differences involving the posterior DMN, Schumacher and colleagues proposed that fMRI‐based functional connectivity is an adequate measure to assess pathology spread across the neural network in DLB.^48^ Specifically, they showed that a higher functional connectivity was accompanied by a higher covariance of tau uptake in PET and, further, that functional connectivity to tau hot and cold spots predicted high and low tau accumulation, respectively, in the connected region. Functional connectivity findings also closely align to the neural structure as evidenced by a single study that constructed GMNs based on correlations of cortical thickness in DLB (S5 in Appendix S1; Fig. 3B), showing disruptions primarily in the DMN regions.^49^

Functional as opposed to structural MRI‐based connectivity may be closely aligned with the characteristic cognitive fluctuations observed in DLB. Yet, it can be argued that the static correlations assessed by most of the studies neglect the variability of functional connectivity measures over time, which may be of particular interest to fully appreciate the transient nature of symptoms in DLB.

Dynamic functional connectivity analyses^50^, ^51^ allow the evaluation of connectivity changes over time. So far, three studies have used dynamic functional connectivity analyses in DLB^41^, ^52^, ^53^ to show that patients with DLB spend more time in states with overall sparser^52^ and less‐positive correlations.^53^ However, these prolonged dwelling times in more segregated states may only emerge at later disease stages because Schumacher and colleagues did not find analogous results in prodromal DLB cases.^54^

### 2.4 Positron Emission Tomography

FDG‐PET can be used to characterize the regional glucose consumption as a marker of neuronal activity. Networks of metabolic connectivity between distinct brain regions can be constructed based on the covarying PET signal between brain areas (S7 in Appendix S1; Fig. 3D).

Coinciding with the findings of structural disconnections in DLB, four studies using graph‐theoretical analyses on FDG‐PET revealed substantial connectivity differences and a disorganization of the network structure in patients with DLB compared with HCs.^10^, ^55^, ^56^, ^57^ Caminiti and colleagues^55^ found a lower clustering coefficient and modularity in patients, indicating a less segregated network in DLB compared with HCs. Concurrently, a greater global efficiency and shorter characteristic path length in patients suggested a higher integration in the DLB group. Taken together, these results indicate a disorganization and dedifferentiation in the network structure of patients with DLB. In addition, Chen and colleagues reported a loss of small‐worldness features in the neural networks of patients with DLB, as reflected by a decreased clustering coefficient and an increased characteristic path length.^10^ Furthermore, global and local efficiency were decreased in their patients with DLB. The study also uncovered a distinct rightward asymmetry of local and global efficiency in the DLB group, suggesting a less effective communication within the left hemisphere. Similarly, Imai and colleagues found a decrease in global connectivity in patients with DLB compared with HCs.^57^ The most remarkable differences emerged in the right posterior cingulate and the transverse temporal gyrus. Considering their involvement in the DMN, this finding once more corroborates the importance of the DMN in DLB.

In this vein, two studies investigated the FDG‐PET–based connectivity within and between several resting‐state brain networks defined by fMRI.^11^, ^58^ In the study by Sala and colleagues, within‐network disruptions predominantly emerged in the primary visual network (VN), limbic network, and posterior DMN.^58^ In addition, between‐network disruptions converged on the primary VN and DAN.

Alongside the interest in functionally defined resting‐state networks, two studies from the same research group demonstrated severe impairments in FDG‐PET networks defined by their predominant neurotransmitters.^12^, ^55^ The cholinergic networks, with the exception of the cholinergic Ch1‐Ch2 division networks, were severely disrupted in DLB.^12^ The importance of the cholinergic system was also underlined by the only SPECT‐based connectivity study so far^59^ that identified a pattern of cholinergic M_1_/M_4_ receptor expression that differentiated patients with DLB from HCs (S8 in Appendix S1; Fig. 3E).

Moreover, the noradrenergic network was shown to undergo a complete reconfiguration in DLB.^12^ Likewise, the two FDG‐PET^12^, ^55^ studies demonstrated that DLB is associated with extended metabolic connectivity alterations in the nigro‐striato‐cortical dopaminergic network. This led the authors to argue that DLB, and synucleinopathies in general, can be regarded as multisystem disorders. Huber and colleagues zoomed in even closer into the association between dopaminergic system and FDG‐PET–based connectivity.^13^ Stratified by dopamine degeneration, the FDG‐PET connectivity differed across stages of nigrostriatal degeneration: in patients with mild dopamine deficiency, connectivity was higher in the basal ganglia and the limbic system, whereas connectivity was lower in patients with more pronounced dopamine deficiency.

### 2.5 Electroencephalography

In the field of EEG, connectivity builds on correlations between the time series of neural oscillations. Compared with the other methods in this review, connectivity in EEG exhibits a lower spatial resolution. On the other hand, EEG provides a higher temporal resolution and allows tapping into different frequency domains of neural oscillations (S9 in Appendix S1).

Across the different methods that investigated EEG connectivity in DLB, studies reported consistent disruptions in the alpha band in patients with DLB compared with HCs.^60^, ^61^, ^62^, ^63^ In addition, studies also found congruent results of a reduced connectivity in the alpha frequency range at the prodromal DLB stage.^64^ Approaching disturbances in the alpha band from a network point of view, three studies conducted graph‐theoretical analyses.^65^, ^66^, ^67^ Van Dellen and colleagues reported network disruptions in the alpha band in patients with DLB^65^, a finding that was endorsed by two subsequent studies that explored different EEG connectivity measures in the same cohort.^66^, ^67^ Interestingly, Mehraram and colleagues further demonstrated that long‐distance, frontoparietal connections were affected in particular.^67^ Taking it a step further, Dauwan and colleagues explored directed connectivity in DLB.^68^ In contrast to HCs, in whom occipital regions drove activity in the frontal areas in the alpha band, this posterior–anterior gradient was significantly disturbed in patients with DLB. This result mirrors the decreased causal connectivity between the frontal and parietal areas of patients with DLB described in the previous fMRI section.^43^

Although most of the studies summarized in this section conducted their analyses in the delta, theta, alpha, and beta frequency bands, significant differences were mostly limited to the alpha band. That said, disruptions arising in the beta band network typically aligned to those obtained for alpha frequencies, thus emphasizing their common association with attentional processes.^69^ In a study by Kai and colleagues, intrahemispheric and to a lesser degree interhemispheric connectivity were reduced in the beta band in patients who did not take donepezil.^60^ Patients who had undergone donepezil treatment, however, only exhibited a reduction in intrahemispheric connectivity in the beta band between two temporal EEG electrodes (T3–T5). This discrepancy according to medication intake was contradicted by a subsequent study that used a different connectivity measure.^67^ Although the majority of patients with DLB were on cholinesterase inhibitors, beta band connectivity was decreased across all distance ranges.

At theta frequencies, Kai and colleagues revealed a widespread decrease in intrahemispheric connectivity. On the other hand, differences in interhemispheric connectivity were more focalized with only a single pair of temporal electrodes (T3–T4) and a pair of frontal electrodes (F3–F4) showing decreased and increased interhemispheric connectivity, respectively.^60^ Two more studies also evidenced an increased network segregation in the theta band.^66^, ^67^ Only two early studies reported significant connectivity differences in the delta band. Whereas Kai and colleagues found decreased intra‐ and interhemispheric connectivity across multiple electrode pairs,^60^ Andersson and colleagues reported an overall increase in delta band connectivity in patients with DLB.^61^

## 3 Discussion

### 3.1 Congruent Patterns of (Dis‐)Connectivity Across Modalities

**FIG 4:**
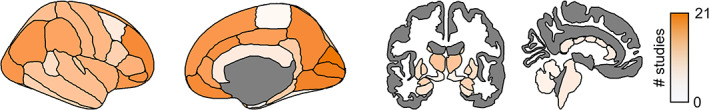
Combined connectivity differences in patients with dementia with Lewy bodies compared with healthy controls across diffusion tensor imaging, gray matter networks, functional magnetic resonance imaging, fluorodeoxyglucose positron emission tomography, and single‐positron emission computer tomography. [Color figure can be viewed at wileyonlinelibrary.com]

This systematic review shows that the neural networks of patients with DLB were predominantly characterized by disruptions in comparison with HCs, as revealed by all the neuroimaging measures covered in this review. Graph‐theoretical analyses on structural and functional MRI, PET, and EEG data evidenced that the brain's global topology, and with this the efficient information transfer, is compromised in DLB. Long‐distance connections, especially cholinergic connections,^60^, ^70^ seem to be particularly affected by disruptions. We found that WM disruptions in DLB are severe and extend to more brain regions than in patients with Parkinson's disease dementia.^19^ In contrast, the network disruptions are less diffuse compared with those in patients with Alzheimer's disease.^57^ Across techniques, the more severely affected brain regions in DLB converged on the FPN, DMN, and VNs (Fig. 4).

Neural networks are small‐world networks laid out to balance an efficient transfer and integration of information with the costs to maintain the network.^71^ Any large deviation from this small‐worldness in either direction indicates a disequilibrium. Therefore, the ostensibly higher network efficiency and connectivity of patients with DLB identified by some studies^40^, ^45^, ^46^, ^55^ may indicate a sparing of short‐range relative to long‐range connections. Furthermore, this finding may indicate an initial compensatory process that may eventually overburden the neural system.^37^ Because the stability of graph‐theoretical measures depends on the sample size,^72^ these findings should be interpreted with caution. Yet, despite some heterogeneous results within any single imaging modality, complementing results across distinct methods reinforce their validity in improving our understanding of DLB.

### 3.2 Connectivity Measures and Clinical Phenotype

The convergence of abnormal connectivity detected in the FPN, DMN, and VNs across imaging techniques speaks for the validity of these findings. The importance of the three networks is further corroborated when taking into account their involvement in cognitive functions typically affected in DLB, that is, attention, visuospatial abilities, and executive functions.^73^ For example, disconnections in the FPN have been associated with cognitive fluctuations,^37^ a core clinical feature of DLB. The VN and DMN have been implicated in VHs, another core clinical feature of DLB.^74^ In addition, disruptions in the DMN and VNs, as observed in fMRI and PET, overlap with the loss of connectivity in the alpha network in EEG, which similarly denote impairments in visual attention.^75^

Although our review process was not tailored to capture studies investigating associations between neural connectivity and clinical phenotype, a subset of the reviewed studies probed these associations, mainly focusing on VHs, cognition, and parkinsonism. For example, disruptions of the ILF were negatively correlated with the severity and frequency of VHs and adversely affected visual attention.^24^, ^30^ Using FDG‐PET, Iaccarino and colleagues showed that the location of the most marked differences within and between the VNs, DMN, ventral attention network, and DAN relate to the presence or absence of VHs.^11^ The involvement of the VN in VHs was confirmed in a study by Sala and colleagues.^58^ Concerning the coherence in EEG bands, a reduced connectivity strength in the alpha band correlated with VH scores, thus supporting the role of alpha oscillations in attention and their link to VHs.^67^ Likewise, a diminished coupling of theta oscillations was related to VHs.^66^

In addition, several studies^18^, ^20^ showed a correlation between WM integrity and cognitive function, a relationship that extended to the intactness of the alpha band network. Precisely, reduced intra‐ and interhemispheric connectivity in the alpha band was accompanied by worse cognitive performance in various tests.^63^, ^64^, ^65^ However, the correlation between alpha network characteristics and clinical variables was not confirmed by another study.^66^ In addition to global cognitive decline, DLB is characterized by cognitive fluctuations, which have been associated with disruptions in frontoparietal connections.^37^, ^43^ Nevertheless, depending on the brain region, both decreases and increases in functional connectivity have been implicated in cognitive fluctuations.^40^ Similarly, in a study by Peraza and colleagues,^47^ greater regional homogeneity in the bilateral cuneus was accompanied by higher cognitive fluctuation scores.

Regarding motor symptoms, WM disruptions in the fornix^31^ corresponded to the severity of motor symptoms. In contrast, higher correlations in the basal ganglia and limbic networks have been related to parkinsonism and mood disturbances.^44^ However, it has been discussed that the association of motor disturbances with the limbic system is not direct, but perhaps caused by their co‐occurrence with affective symptoms.^76^, ^77^

Despite the described associations between connectivity disruptions and DLB core clinical features, these associations did not consistently materialize in all studies.^27^, ^29^, ^35^, ^39^, ^59^ This may be attributable to the diverse range of symptoms in patients with DLB, since the presence of two core clinical features or the combination of one core clinical feature and one indicative biomarker merit a diagnosis of probable DLB according to current diagnostic criteria.^2^ Although a few studies tried to counter this heterogeneity by screening their patient cohort for one specific clinical feature,^21^ the small number of patients with DLB (n ≤ 25) and the varied manifestation of the disease may explain the diversity of findings. Hence, guiding the choice of ROIs and/or seed regions by the core clinical features of DLB may contribute to more congruent results. The recent emergence of global consortia^78^ and their promotion of large multicenter studies will boost the power of future studies to examine the associations between imaging measures and the clinical features of DLB.^79^, ^80^, ^81^ We thus envision that some of these associations will be elucidated in the coming years.

### 3.3 Clinical Implications and Connectivity Measures Along the Progression of the Disease

Although the investigation of connectivity, as opposed to regional differences in volume and functions, has only recently picked up pace, the studies summarized in this systematic review indicate that connectivity measures have the potential to become suitable biomarkers for DLB in the future.

Although not explicitly formulated as connectivity measures, FDG‐PET and EEG already have a place as supportive biomarkers in the current diagnostic criteria for DLB.^2^ Likewise, the recent diagnostic criteria for prodromal DLB list FDG‐PET and EEG as potential biomarkers.^16^

Several studies^27^, ^29^, ^30^, ^32^ found positive correlations between the integrity of WM tracts and cortical thickness in the connected brain regions, suggesting an at least equivalent diagnostic value of connectivity measures compared with conventional measures. A study demonstrated that brain regions with altered WM properties overlapped with areas showing glucose hypometabolism,^21^ illuminating the link between network structure and function. Especially in the early stages of the disease, connectivity measures in DLB may not only mirror but also provide ancillary information to volumetric measures. This is supported by studies^22^, ^24^ that showed that WM differences emerged in the absence of detectable gray matter (GM) atrophy. To reconstruct in which order WM and GM alterations appear in the disease process of DLB and to which extent one drives the other, longitudinal assessments are necessary. Nevertheless, the available literature already speaks for a clinically valuable sensitivity of WM disruptions to changes early in the disease progress. Indeed, disruptions in NBM WM projections were superior to NBM volume in predicting the conversion from mild cognitive impairment (MCI) to DLB.^33^ Although the current research criteria for prodromal DLB^16^ emphasize the predictive power of quantitative EEG, connectivity measures remain regretfully underappreciated notwithstanding that differences to HCs were already detectable at the MCI stage in many measures presented in this review. Early disruptions occurred in oscillatory networks.^64^ Likewise, patients at the mild dementia stage spent more time in a sparser connected state compared with HCs.^41^ In addition, Huber and colleagues^13^ observed an increase in metabolic connectivity at the early disease stages, which was interpreted as a compensatory process to counter the beginning of dopamine deficiency. With increasing dopamine deficiency in later disease stages, metabolic connectivity breaks down, possibly indicating advanced neurodegeneration. Although these results suggest that network alterations can be adduced as early biomarkers in DLB, they also indicate that alterations may be difficult to capture when measurements occur around a putative inflection point, thus providing a possible explanation for the null findings by some authors.^54^

So far, the evidence on longitudinal connectivity changes is very limited, as the only longitudinal study to date did not detect any WM changes accruing in a year.^22^ As 1 year is a relatively short time period, longer follow‐ups are needed to establish whether connectivity changes continue to accrue over time. In the future, a greater number of longitudinal studies covering different imaging modalities, combined with postmortem assessments of cortical Lewy body pathology, will be indispensable to characterize the progression of the neurodegenerative process driven by α‐synuclein–related pathology in DLB. Multimodal longitudinal studies that combine assessments of structural and functional connectivity will be of particular interest to disentangle the complex relationship between structural and functional neural changes in the disease progress of DLB. The knowledge of whether early functional changes drive structural changes or initial structural changes lead to the deterioration of function will allow us to gauge potential compensatory effects and how those may be exploited for treatment purposes.

Connectivity measures were sensitive to treatment effects inasmuch as patients not receiving donepezil exhibited more widespread disruptions in the beta band network than patients taking the medication.^60^ In addition, Colloby and colleagues^59^ probed the cholinergic M_1_/M_4_ receptor after 12 weeks of donepezil treatment. They revealed distinct binding patterns that correlated with improvements in cognitive and VH scores. This suggests that the value of tracing changes in the cholinergic network in DLB is not restricted to reconstructing the disease progress possibly from the early stages onward. Instead, the integrity of the cholinergic network may be adduced to predict and monitor treatment response. These studies not only imply that the intake of medication may have obscured more consistent findings but also they point to the necessity of stratifying patients in terms of their medication to render a systematic characterization possible.

### 3.4 Limitations

In this systematic review, we attempted to cover all relevant literature on the topic of connectivity in DLB. Meta‐analyses can provide a quantitative summary of the existing literature, but the heterogeneity in imaging modalities and analytic procedures within modalities made this approach unfeasible.

The JBI criteria indicated that some methodological issues should be considered. In particular, PET‐based connectivity studies tended to be classified into the low‐quality category of the JBI because of the lack of information about the HCs or not completely taking demographical group differences into account. Moreover, most PET studies did not correct for motion artifacts or signal decay, which may have partly interfered with quantitative accuracy.^82^, ^83^ However, the results of the PET studies are largely consistent with each other and the results of other imaging modalities, which encouraged us to retain them as a relevant part of the reviewed literature.

Likewise, the impact of patients' medications on connectivity measures should be further investigated. The results on the impact of acetylcholinesterase inhibitors on connectivity measures vary.^42^, ^60^, ^66^, ^67^ In addition, this review showed that antiparkinson agents, antipsychotics, and antidepressants are also commonly prescribed to patients with DLB, and their effects on connectivity measures should be considered in future research.

This review highlights the need for more connectivity studies in DLB, especially in currently underrepresented modalities such as GMNs and SPECT. Moreover, the value of other imaging modalities in assessing connectivity differences between patients with DLB and HCs is still uncharted, for example, magnetoencephalography (MEG). Its relatively high spatial and temporal resolution makes MEG a promising tool to uncover connectivity differences between patients with DLB and HCs. In addition, other analysis techniques can be applied to outline abnormal neural patterns. Among those, principal component analysis was previously used to reveal metabolic patterns in FDG‐PET in patients with DLB.^84^, ^85^

Aside from the sensitivity to disease‐related aspects in DLB, connectivity measures also need to demonstrate specificity to be considered a valuable biomarker for DLB. So far, only two EEG‐based connectivity studies provided details on classification accuracy when differentiating HCs from DLB and prodromal DLB, reaching a moderate classification accuracy in both cases.^63^, ^64^ Given the heterogeneous clinical phenotype of DLB, the moderate classifier performance should not be neglected. This also underscores the potential need for adjusting cutoffs according to the patient's clinical presentation in addition to exploring the discriminatory power of connectivity measures in other imaging modalities.

## 4 Conclusion

Congruent findings across distinct imaging modalities testify to the value of assessing connectivity in DLB. Which of the discussed methods provides the best predictive value and aligns more closely with clinical phenotype in DLB cannot be established at present because direct comparisons between imaging modalities are still missing. In addition, it should be considered that the various imaging modalities catch different time scales. Although DTI and PET are appropriate tools for the assessment of a trait, the high temporal resolution of EEG qualifies it for assessing a state. Gauging the disease state may be particularly important in DLB, which is characterized by cognitive fluctuations on different time scales. Although parallels emerged between structural and functional disintegration in DLB, more studies are needed to probe the structural basis of functional networks in DLB in greater depth. Apart from collecting multimodal data, future studies should place more emphasis on acquiring data in larger and better characterized patient populations, factoring in brain state, symptomatology, and medication use of individual patients. This development is facilitated by the recent establishment of large multicenter cohorts in different national and international consortia.^78^

In conclusion, connectivity measures show great potential to capture brain disruptions in patients with DLB, possibly before regional volumetric differences can be observed. Tracing connectivity disruptions may be of particular importance given the often limited level of atrophy encountered in DLB, especially in its prodromal stage.^86^

## Author Roles

(1) Research Project: A. Conception, B. Organization, C. Execution; (2) Manuscript: A. Writing of the First Draft, B. Review and Critique.

A.H.: 1A, 1B, 1C, 2A, 2B

L.‐O.W.: 2B

E.W.: 2B

T.D.: 2B

D.F.: 1A, 1B, 2B

### Financial Disclosures

A.H. received funding from the Stohnes Foundation, Demensfonden, Loo och Hans Osterman Stiftelse (2022‐01218), and Foundation for Geriatric Diseases at Karolinska Institutet (2022‐01274). E.W. reports grants from the Swedish Research Council (2021–01861), the Swedish Foundation for Strategic Research (SSF, RB13‐0192), the Swedish State under the agreement between the Swedish government and the county councils, the ALF agreement (FoUI‐954,893), the Center for Medical Innovation (CIMED, FoUI‐954,459), the Swedish Alzheimer Foundation (AF‐939687), and the Swedish Brain Foundation (FO2021‐0119). D.F. received funding from the Center for Innovative Medicine (CIMED, 20200505), the regional agreement on medical training and clinical research (ALF, FoUI‐962,240) between Stockholm County Council and Karolinska Institutet, Hjärnfonden (FO20200066), Alzheimerfonden (AF‐968032), Demensfonden, Neurofonden, Stiftelsen För Gamla Tjänarinnor, and Gun och Bertil Stohnes Stiftelse. L.‐O.W. and T.D. have nothing to disclose.

## Supporting information


**Appendix S1** Supporting information

## Data Availability

Data sharing is not applicable to this article as no new data were created or analyzed in this study.
