# Supplementary material for: (Dis‐)Connected Dots in Dementia with Lewy Bodies—A Systematic Review of Connectivity Studies
Source: Mov Disord. 2022 Oct 17;38(1):4–15. doi: 10.1002/mds.29248 (PMC10092805; doi:10.1002/mds.29248)
Supplement: Supplementary file 1 — Appendix S1 Supporting information [file MDS-38-4-s001.pdf]

Supplementary Material

S1 Full search strings for the three literature data bases.

PubMed

((dementia with lewy bodies[Title/Abstract]) OR (lewy body dementia[Title/Abstract])) AND (((connecti*[Title/Abstract]) OR (network[Title/Abstract])) OR (graph*[Title/Abstract]))

Scopus

(TITLE-ABS-KEY (dementia AND with AND lewy AND bodies) OR TITLE-ABS-KEY (lewy AND body AND dementia) AND TITLE-ABS-KEY (connecti*) OR TITLE-ABS-KEY (network) OR TITLE-ABS-KEY (graph*))

Web of Science

((TS=(dementia with lewy bodies)) OR TS=(lewy body dementia)) AND ((TS=(connecti*)) OR (TS=(network)) OR (TS=(graph*)))

Both “dementia with Lewy bodies” and “Lewy body dementia” were included as search terms, since earlier studies used the two interchangeably, but screening was designed to only select DLB studies.

S2 Overlap between studied DLB cohorts across international centres.

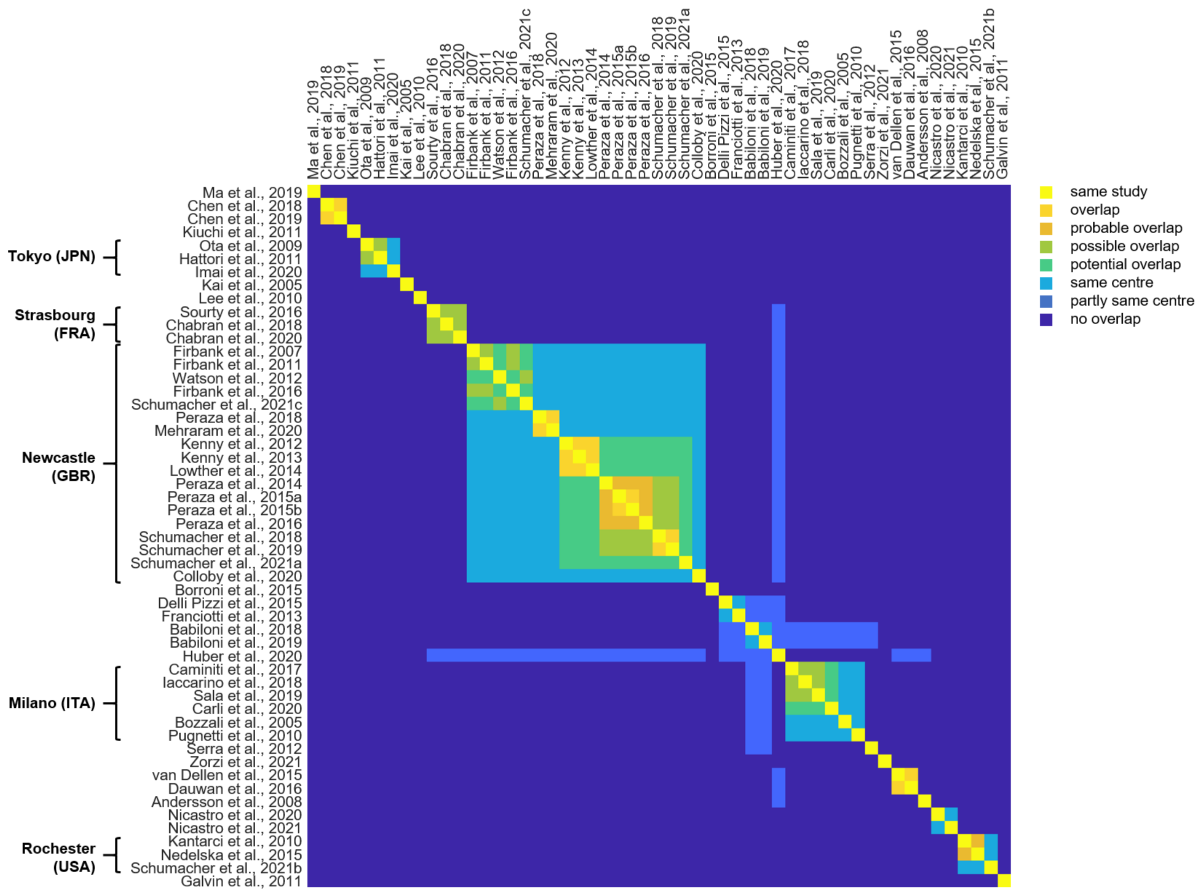

Table 1 Quality of included studies.

S3 Quality of included studies.

**Publication**	Q1	Q2	Q3	Q4	Q5	Q6	Q7	Q8	Q9	Q10	Total	Quality
Andersson et al., 2008 [62]	1	1	1	1	1	0	1	0	1	0	7	moderate
Babiloni et al., 2018 [64]	1	1	1	1	1	0	1	1	1	1	9	high
Babiloni et al., 2019 [65]	1	1	1	1	1	1	1	1	1	1	10	high
Borroni et al., 2015 [35]	0	1	1	1	1	0	0	1	1	1	7	moderate
Bozzali et al., 2005 [18]	1	0	1	1	1	0	1	0	1	0	6	low
Caminiti et al., 2017 [56]	1	1	1	1	0	0	1	1	1	1	8	moderate
Carli et al., 2020 [12]	0	1	1	1	1	0	1	0	1	1	7	moderate
Chabran et al., 2018 [40]	0	1	1	1	1	0	1	0	1	0	6	low
Chabran et al., 2020 [41]	1	1	1	1	1	0	1	0	1	0	7	moderate
Chen et al., 2018 [10]	0	1	1	1	0	0	0	0	1	1	5	low
Chen et al., 2019 [57]	0	1	1	1	0	0	0	0	1	1	5	low
Colloby et al., 2020 [60]	1	1	1	1	0	0	1	0	1	1	7	moderate
Dauwan et al., 2016 [69]	1	1	1	1	1	1	1	1	1	1	10	high
Delli Pizzi et al., 2015b [33]	1	0	1	1	0	1	1	0	1	0	6	low
Firbank et al., 2007 [23]	1	0	1	1	1	0	1	1	1	0	7	moderate
Firbank et al., 2011 [31]	1	1	1	1	1	1	1	0	1	1	9	high
Firbank et al., 2016 [22]	0	1	1	1	1	0	1	1	1	0	7	moderate
Franciotti et al., 2013 [44]	1	1	1	1	1	1	1	0	1	0	8	moderate
Galvin et al., 2011 [43]	0	1	1	1	1	1	1	0	1	0	7	moderate
Hattori et al., 2011 [27]	1	1	1	1	1	0	1	1	1	1	9	high
Huber et al., 2020 [13]	0	1	1	0	0	0	1	0	1	1	5	low
Iaccarino et al., 2018 [11]	1	1	1	0	0	0	1	0	1	1	6	low
Imai et al., 2020 [58]	0	1	1	1	1	0	0	0	1	1	6	low
Kai et al., 2005 [61]	0	0	0	1	1	0	0	0	1	1	4	low
Kantarci et al., 2010 [24]	1	1	1	1	1	0	1	0	1	0	7	moderate
Kenny et al., 2012 [46]	1	1	1	1	1	0	1	0	1	0	7	moderate
Kenny et al., 2013 [47]	1	1	1	1	1	0	1	0	1	0	7	moderate
Kiuchi et al., 2011 [28]	1	1	1	1	1	0	1	0	1	0	7	moderate
Lee et al., 2010 [19]	1	1	1	1	1	1	1	0	1	0	8	moderate
Lowther et al., 2014 [45]	1	1	1	1	1	0	1	0	1	0	7	moderate
Ma et al., 2019 [54]	1	1	1	1	1	0	1	0	1	0	7	moderate
Mehraram et al., 2020 [68]	1	0	1	1	1	0	1	1	1	1	8	moderate
Nedelska et al., 2015 [21]	1	1	1	1	1	0	1	1	1	0	8	moderate
Nicastro et al., 2020 [32]	1	1	1	1	1	0	1	0	1	1	8	moderate
Nicastro et al., 2021 [50]	1	1	1	1	1	0	1	0	1	1	8	moderate

Ota et al., 2009 [25]	1	1	1	1	1	0	1	0	1	1	8	moderate
Peraza et al., 2014 [38]	1	1	1	1	1	0	1	0	1	1	8	moderate
Peraza et al., 2015a [37]	1	0	1	0	1	0	1	0	1	0	5	low
Peraza et al., 2015b [39]	1	0	1	1	1	0	1	0	1	1	7	moderate
Peraza et al., 2016 [48]	1	0	1	1	1	0	1	0	1	0	6	low
Peraza et al., 2018 [67]	1	0	1	1	0	0	1	1	1	1	7	moderate
Pugnetti et al., 2010 [63]	0	1	1	1	0	1	0	1	1	1	7	moderate
Sala et al., 2019 [59]	0	1	1	1	0	0	1	1	1	0	6	low
Schumacher et al., 2018 [36]	1	1	1	1	1	0	1	0	1	0	7	moderate
Schumacher et al., 2019 [53]	1	1	1	1	1	0	1	0	1	0	7	moderate
Schumacher et al., 2021a [55]	0	1	1	1	1	1	1	0	1	1	8	moderate
Schumacher et al., 2021b [49]	1	1	1	1	1	0	1	0	1	0	7	moderate
Schumacher et al., 2021c [34]	1	1	1	1	1	0	1	0	1	0	7	moderate
Serra et al., 2012 [26]	0	1	1	1	1	0	1	0	1	0	6	low
Sourty et al., 2016 [42]	0	0	0	1	1	0	0	1	1	1	5	low
van Dellen et al., 2015 [66]	1	1	1	1	1	0	1	1	1	1	9	high
Watson et al., 2012 [20]	1	1	1	1	1	0	1	1	1	1	9	high
Zorzi et al., 2021 [30]	1	0	1	1	1	1	1	1	1	1	9	high

Criteria based on 10-item checklist for case-control studies developed by the JBI Collaboration [8]. Q1: Were the groups comparable other than presence of disease in cases or absence of disease in controls? Q2: Were cases and controls matched appropriately? Q3: Were the same criteria used for identification of cases and controls? Q4: Was exposure measured in a standard, valid and reliable way? Q5: Was exposure measured in the same way for cases and controls? Q6: Were confounding factors identified? Q7: Were strategies to deal with confounding factors stated? Q8: Were outcomes assessed in a standard, valid and reliable way for cases and controls? Q9: Was the exposure period of interest long enough to be meaningful? Q10: Was appropriate statistical analysis used?

S4 DTI-based connectivity in DLB.

**Authors (year)**	**DLB patients (F:M) age MMSE Diagnosis criteria Medication** [1)]	**HC (F:M) age MMSE** [1)]	**Other patient groups**	**Additional measures**	**Acquisition Details**	**Methods**	**Connectivity results** [2)]	**Correlations with other measures**
Bozzali et al. (2005) [18]	15 (8:7)<br>77.1 ± 5.1<br>MMSE: 22.7 [9.3 – 26.7]<br>Diagnosis criteria: McKeith et al., 1996<br>Medication: N/P	10 (6:4)<br>75.3 [69-82]<br>MMSE: 28.6 [26.8 – 30.0]		VBM	1.5 T Siemens,	rectangular ROIs (genu and splenium of CC, posterior limb and genu of internal capsule, anterior/posterior pericallosal areas, WM of frontal, parietal, temporal and occipital lobes, thalamus, putamen, head of caudate nucleus), FA and MD	- MD ↑ in frontal, parietal, and occipital WM, CC, pericallosal areas and caudate nucleus<br>- FA ↓ in frontal, parietal, occipital, and temporal WM, CC and pericallosal regions	- predicting model for dual performance test with MD of frontal WM<br>- predicting model for phonemic, categorical fluency with FA of frontal WM and<br>- predicting model for fragmented letter subtest with FA of temporal WM<br>- predicting model for size discrimination with MD of occipital WM<br>- predicting model for shape and size discrimination, constructional praxis ability test with FA of parietal WM
Delli Pizzi et al. (2015) b [33]	14 (7:7)<br>75.8 ± 3.8<br>MMSE: 18.0 ± 4.9	15 (8:7)<br>75.0 ± 4.8	AD	VBM, clinical and neuro	3.0 T Philips Achieva	18 tracts: corticospinal tract, ILF, UF, ATR,	- DTI-metric changes in r ATR (DA), r ILF (FA), left CCG (DR), r (DR, MD) and l (DA, DR, MD) UF	- +corr between DA in right anterior thalamic radiation to MD in r thalamus

Front. Aging Neurosci.	Diagnosis criteria: McKeith et al., 2005 Medication: All patients on L-DOPA and ChEI, 5 on quetiapine, 4 on clozapine, 14 on clonazepam .	MMSE: 27.7 ± 0.6		psychological assessments		CCG, CAB, superior longitudinal fasciculus-parietal bundle, superior longitudinal fasciculus-temporal bundle, CC-forceps major, and CC-forceps minor, FA, MD, DA, and DR		- –corr between DR in l CCG bundle and cortical thickness in l precuneus<br>- –corr between DA in l uncinate fasciculus and cortical thickness in l mediorbitofrontal gyrus, l laterorbitofrontal gyrus, l pars triangularis, l parahippocampus<br>- –corr DR in l uncinate fasciculus and cortical thickness in l laterorbitofrontal gyrus and l pars triangularis<br>- n.s. to between DTI-metrics and cortical thickness in non-target regions
Firbank et al. (2007) b [23]	16 (7:9) 76.0 ± 7.0 MMSE: 19.1 ± 4.5 Diagnosis criteria: McKeith et al., 1996 Medication: 9 patients on ChEI.	15 (6:9) 75.0 ± 8.0 MMSE: 28.3 ± 2.1	AD		1.5 T Intera Philipps	ROIs (putamen, head of caudate, genu and splenium of CC, anterior/posterior pericallosal area, parietal, frontal, occipital, and temporal WM, anterior/posterior internal capsule, thalamus), FA and ADC	- FA ↓ in precuneus	

Firbank et al. (2011) [31]	16 (6:10)<br>81.0 ± 5.9<br>MMSE: [18, 15-27]<br>Diagnosis criteria: McKeith et al., 2005<br>Medication: 9 patients on anti-parkinson medication.	16 (7:9)<br>76.3 ± 8.2<br>MMSE: [29, 26-30]	AD	VBM	3.0 T Intera Achieva	ROI in fornix, FA	- FA ↓ in WM of postcentral gyrus (28, -28,48)	- corr between hippocampus visual rating and FA in l/r periventricular WM, l/r CC body, r anterior frontal lobe, fornix, stria terminalis, r superior cerebellar peduncle (across groups)<br>- -corr between FA and UPDRS motor score (in DLB and combined dementia groups)
Firbank et al. (2016) [22]	14 (1:13)<br>77.2 ± 8.0<br>MMSE: 21.2 ± 6.0<br>Diagnosis criteria: McKeith et al., 2005<br>Medication: N/P.	32 (13:19)<br>76.6 ± 5.3<br>MMSE: 29.2 ± 0.9	AD	Follow-up DTI (after 1 year), VBM	3.0 T Intera Achieva	Whole brain, FA and MD	- n.s. in FA and MD	- corr between brain volume decrease and MD change in whole cohort but not in DLB
Hattori et al. (2012) [27]	29 (13:16)<br>79.1 ± 5.0<br>MMSE: 20.7 ± 6.8<br>Diagnosis criteria:	40 (22:18)<br>76.9 ± 4.9<br>MMSE:	PD, PD-MCI, PDD	VBM, SPECT	1.5 T, Symphony Siemens	Whole brain, FA	- FA ↓ in SLF, ILF, IFOF, UF, cingulum, CC	- no corr between FA values and MMSE scores in DLB

	McKeith et al., 2005<br>Medication: N/P.	29.0 ± 0.8						
Kantarci et al. (2010) [24]	30 (4:26)<br>[71, 55-85]<br>MMSE: N/P<br>Diagnosis criteria: McKeith et al., 2005<br>Medication: N/P.	60 (10:50)<br>[73, 54-86]<br>MMS: N/P	AD	Clinical assessments	3.0 T, GE	ROIs according to AAL (amygdala, hippocampus, PHG, PCC, precuneus, frontal, parietal, occipital, temporal lobes; pre/postcentral gyrus as reference) and specific tracts (fornix, corticopontine tracts, ILF, SLF, cingulum bundles, CC), MD and FA	- FA ↓ and MD ↑ in ILF	- +corr between MD and UPDRS scores<br>- higher MD in ILF only in DLB with VHs<br>- addition of FA measures to GM density improved distinction between AD and DLB/HC only in hippocampus and PHG
Kiuchi et al. (2011) [28]	26 (20:6)<br>74.2 ± 7.4<br>MMSE: 19.1 ± 4.3<br>Diagnosis criteria:	26 (17:9)<br>71.4 ± 7.4<br>MMSE:	AD		1.5 T Sonata Siemens	Tractography of UF, IFOF, ILF,<br>FA	- FA ↓ for l/r UF, l/r IFOF, and l ILF	

	McKeith et al., 2005<br>Medication: 11 pateints on ChEI, 7 on anti-parkinson medication.	28.5 ± 1.7						
Lee et al. (2010) [19]	18 (13:5)<br>75.2 ± 7.1<br>MMSE: 14.6 ± 4.5<br>Diagnosis criteria: McKeith et al., 2005<br>Medication: 5 patients on ChEI, 7 on dopaminergic medication.	18<br>71.2 ± 6.5<br>MMSE: 28	PDD		3.0 T Philips Intera	Whole brain WM, FA	- FA ↓ l/r orbitofrontal, anterior and middle portion of cingulate, r dlPFC, l anterior temporal, l parietal, l/r temporal, l/r insular, l PCC, l/r visual association regions	
Nedelska et al. (2015) [21]	30 (5:25)<br>[69, IQR: 63-76]<br>MMSE: [20.5, IQR: 15-24]<br>Diagnosis criteria:	60 (10:50)<br>[68.5, IQR: 63-76]<br>MMSE:	AD	FDG-PET, PiB-PET	3.0 T, GE	Whole brain, FA	- FA ↓ in posterior parietal and occipital WM (no substantial difference after adjustment for Aβ-load)	- Overlap between glucose hypometabolism and FA↓

	McKeith et al., 2005 Medication: N/P.	[29, IQR: 28-29]						
Nicastro et al. (2020) [32]	19 (4:15) 73.5 ± 6.1 MMSE: 22.5 ± 4.3 Diagnosis criteria: McKeith et al., 2005; 2017 Medication: N/P.	20 (10:10) 71.0 ± 6.9 MMSE: 28.8 ± 1.2		VBM, PET	3.0 T	Whole brain	- MD and DR ↑ in body and splenium of CC<br>- FA ↓ in body and splenium of CC<br>- FA ↓ and DR ↑ in r anterior and posterior corona radiata, l cingulate gyrus, r SLF (driven by PiB+ subgroup)<br><br>- GM ↓ in bilateral superior and middle frontal, middle and posterior temporal, inferior parietal and lateral occipital cortices	- -corr between ^{11}C-PK11195 uptake and MD and RD in bilateral corona radiata, posterior thalamic radiations, saggital striatum, l SLF, frontal, and parieto-occipital WM tracts
Ota et al. (2009) [25]	14 (5:9) 74.5 ± 6.4 MMSE: N/P Diagnosis criteria: McKeith et al., 2005 Medication: N/P.	13 (5:8) 72.5 ± 4.3 MMSE: N/P			1.0 T Siemens Harmony	Whole brain and specific tracts (splenial fibre tract, ILF, visual pathway), FA and MD	- ↓ FA in ILF	

Schumacher et al. (2021) c [34] *Brain*	48 (13:35) 76.2 ± 6.6 MMSE: 22.6 ± 4.5 Diagnosis criteria: McKeith et al., 2017 Medication: 21 patients on anti-parkinson medication.	71 (20:51) 75.3 ± 6.9 MMSE: 28.9 ± 1.0	AD, LB-MCI, AD-MCI	VBM, EEG (for subset)	3.0 T Intera Achieva	5 ROIs (NBM, cingulum, external capsule, anterior commissure, brain stem), NBM mask as seed region, cingulum and external capsule as waypoint masks (only tracts passing through them were retained), MD, DA, and DR	- MD ↑ along medial NBM pathway (only after removing covariate of MD from WM control mask)<br>- MD ↑ along lateral pathway<br>- MD n.s. in cingulum and external capsule	- -corr between NBM pathway diffusivity and NBM volume and MD from WM control mask<br><br>- Higher MD in medial and lateral NBM WM tracts associated with increased risk of dementia onset<br><br>- MD in lateral NBM tracts predicted MMSE scores beyond age<br><br>- Choice reaction time predicted by MD of lateral NBM tract<br><br>- EEG dominant frequency predicted MD in lateral NBM<br><br>- UPDRS score associated with MD of lateral NBM tract (in DLB and LB-MCI)
Serra et al. (2012) [26]	14 (4:10) 74.1 ± 7.4 MMSE: 17.7 ± 2.5 Diagnosis criteria: McKeith et al., 2005 Medication: N/P.	13 (4:9) 64.7 ± 9.2 MMSE: 29.0 ± 2.2	AD, aMCI	Cognitive assessments	3.0 T Siemens Allegra	UF, FA	- FA ↓ in UF	- across all groups mean FA of l uncinate fasciculus predicted phonological verbal fluency
Watson et al. (2012) [20]	35 (8:27) 78.4 ± 6.9 MMSE:	35 (15:20)	AD	Cognitive and	3.0 T Intera Achieva	Whole brain WM tracts, FA and MD	- FA ↓ in precuneal and cingulate gyri as well as in posterior	- -corr between MD in l/r PHG and l cingulate gyrus and episodic memory in DLB and AD

	20.3 ± 5.3<br>Diagnosis criteria: McKeith et al., 2005<br>Medication: N/P.	76.7 ± 5.2<br>MMSE: 29.1 ± 1.0		clinical assessments			thalamic radiation region, including the optic radiation<br>- MD ↑ widespread, including areas of brainstem, thalamus, cingulate, temporal, parieto-occipital and frontal lobes	- +corr between FA in l/r frontal, parietal, and subcortical structures and letter fluency in DLB only<br>- Relationship between MD in brainstem, striatum, precuneus, precentral gyrus and CC and letter fluency in DLB<br>- -corr between FA in subcortical structures and precentral gyrus and UPDRS-III scores
Zorzi et al. (2021) [30]	DLB-VH<br>10 (6:4)<br>76.6 ± 7.35<br>MMSE: 23.5 ± 3.2<br><br>DLB-NVH<br>13 (6:7)<br>74.4 ± 5.2<br>MMSE: 26.3 ± 3.5<br><br>Diagnosis criteria: McKeith et al., 2017<br>Medication: 13 patients on ChEI, 3 on antipsychotics, 4 on L-DOPA.	13 (7:6)<br>70.8 ± 10.9<br>MMSE: 29.2 ± 2.1		VBM, fMRI, cognitive and clinical assessments	1.5 T Philips Achieva	Whole brain and tract-based: UF, ILF, IFOF, frontoparietal SLF,<br>FA and MD	- FA ↓ in r tempo-parietal ILF, frontal IFOF, and UF<br>- diffuse MD ↑ in r tempo-parietal ILF, frontal IFOF, UF, and SLF3	- -corr between FA in r SLF3 and severity of VH<br>- +corr between MD in r SLF3, ILF, IFOF and l UF and severity of VH<br>- -corr between MD in r SLF3 and ILF and performance in digit cancellation task

1) Total number of participants. Ratio between females and males. Mean ± standard deviation (SD) of age and MMSE are reported as available. If only median ± standard error or range are available, these numbers are provided in brackets.
2) Results refer to comparison of DLB patients to HCs if not specified otherwise.

Aβ = amyloid β. AD = Alzheimer’s disease. ADC = Apparent diffusion coefficient. AD-MCI = Mild cognitive impairment with Alzheimer’s disease. aMCI = amnestic mild cognitive impairment. ATR = anterior thalamic radiation. CAB = clinical assessment of fluctuations. cingulum-angular bundle. CAF = Clinician Assessment of Fluctuation. CC = corpus callosum. CCG = cingulum-cingulate gyrus bundle. ChEI = acetylcholinesterase inhibitor. +corr = positive correlation. -corr = negative correlation. DA = axial diffusivity. DLB = dementia with Lewy bodies. dlPFC = dorsolateral prefrontal cortex. DR = radial diffusivity. DTI = diffusion tensor imaging. EEG = encephalography. FA = fractional anisotropy. FDG-PET = ^{18F}2-fluoro-deoxy-d-glucose photon emission tomography. fMRI = functional magnetic resonance imaging. GM = grey matter. HC = healthy control. IFOF = inferior fronto-occipital fasciculus. ILF = inferior longitudinal fasciculus. IQR = interquartile range. l = left. LB-MCI = Mild cognitive impairment with Lewy bodies. MD = medial diffusivity. MMSE = Mini Mental State Examination. MRS = magnetic resonance spectroscopy. NBM = nucleus basalis of Meynert. N.s. not significant. NVH = no visual hallucinations. PCC = posterior cingulate cortex. PD = Parkinson’s disease. PDD = Parkinson’s disease dementia. PET = photon emission tomography. PHG = parahippocampal gyrus. PiB-PET = ^{11}C Pittsbourgh compound B photon emission tomography. PD-MCI = Mild cognitive impairment with Parkinson’s disease. r = right. ROI = region of interest. SLF = superior longitudinal fasciculus. SPECT = single photon emission computed tomography. UF = uncinate fasciculus. UPDRS = Unified Parkinson’s Disease Rating Scale. VBM = voxel-based morphometry. VH = visual hallucinations. WM = white matter.

S5 Grey matter networks in DLB.

**Authors (year)**	**DLB patients (F:M) age MMSE Diagnosis criteria Medication** [1)]	**HC (F:M) age MMSE** [1)]	**Acquisition Details**	**Method**	**Connectivity results** [2)]
Nicastro et al. (2021) [50]	24 (4:20)<br>74.3 ± 6.7<br>MMSE: 22.8 ± 4.3<br>Diagnosis criteria: McKeith et al., 2017<br>Medication: N/P.	23 (8:15)<br>72.3 ± 5.7<br>MMSE: 28.9 ± 1.2	3.0 T Siemens Tim Trio, T1-MPRAGE	Pearson's correlation between 68 ROIs (34 per hemisphere), graph-theoretical analyses	- local efficiency ↓<br>- clustering coefficient ↓<br>- modularity ↓<br>- small-worldness ↓<br>- nodal clustering ↓ in r entorhinal and l MTG<br>- closeness centrality ↓ in r rostral middle frontal, l/r inferior frontal, l paracentral, l isthmus and caudal AC, l insula, l superior temporal, r fusiform, r transverse temporal, l/r supramarginal, l/r cuneus, l pericalcarine, r lingual gyri<br>- nodal efficiency n.s.<br>- disrupted modules in DLB (weaker connectivity and exclusion of regions compared to HC)

1) Total number of participants. Ratio between females and males. Mean ± standard deviation (SD) of age and MMSE are reported as available. If only median ± standard error or range are available, these numbers are provided in brackets.
2) Results refer to comparison of DLB patients to HCs if not specified otherwise.

AC = anterior cingulate. DLB = dementia with Lewy bodies. HC = healthy control. l = left. MMSE = Mini Mental State Examination. MTG = middle temporal gyrus. n.s. = not significant. r = right. ROI = region of interest.

S6 fMRI-based connectivity in DLB.

Authors (year)	DLB patients (F:M) age MMSE Diagnosis criteria Medication [1)]	HC (F:M) age MMSE [1)]	Other patient groups	Additional measures	Acquisition Details	Methods	Connectivity results [2)]	Correlations with other measures
Borroni et al. (2015) [35]	13 (6:7)<br>74.2 ± 5.2<br>MMSE: 20.3 ± 6.1<br>Diagnosis criteria: McKeith et al., 2005<br>Medication: N/P.	10 (7:3)<br>62.2 ± 8.0<br>MMSE: N/P	PD, PDD	VBM	1.5 Siemens Symphony	Regional homogeneity, voxel-by-voxel	- ReHo ↓ in posterior and inferior regions (l fusiform gyrus and pons)	
Chabran et al. (2018) [40]	26 (14:12)<br>73.0 ± 9.0<br>MMSE: 24.4 ± 4.0<br>Diagnosis criteria: McKeith et al., 2005<br>Medication: N/P.	22 (12:10)<br>65.5 ± 9.0<br>MMSE: 28.9 ± 1.0	AD	task-fMRI, clinical assessments	3.0 T Siemens Verio, 32 channel head-coil	ROI-to-ROI connectivity, 6 ROIs (2 visuoperceptual, 2 DMN-related, l/r FPN)	- FC ↓ between the visuoperceptual ROIs during rest<br>- FC ↓ between different subsections of anterior DMN-related ROI (mPFC) and posterior DMN-related ROI (PCC) during rest<br>- FC ↓ between subsection of l FPN and different subsections of posterior DMN-related ROI and r FPN-related ROI during rest<br>- FC ↑ between temporal l FPN-related ROI and l visuoperceptual ROI<br>- posterior DMN synchronization coefficient ↓ during visual object and space perception paradigm	- n.s.

Chabran et al. (2020) [41]	79 (40:39)<br>70.1 ± 9.4<br>MMSE:<br>25.6 ± 4.0<br>Diagnosis criteria:<br>McKeith et al., 2005<br>Medication:<br>N/P.	22 (11:11)<br>66.5 ± 7.8<br>MMSE:<br>28.9 ± 0.9	AD	VBM, clinical assessments	3.0 T Siemens Verio, 32 channel head coil	ROI-to-ROI connectivity, 22 ROIs in SA, FPN, DAN, DMN	- FC ↓ within SA, within FPN (r lPFC and r PPC), and between ROIs of FPN and DAN (r lPFC and r IPS)<br>- FC ↑ between mPFC of DMN and and r rPFC of SA and r lPFC of FPN<br>- mean FC ↓ within SA	- $_{+}$corr between FC (l rPFC and l FEF, l anterior insula and r FEF, r anterior insula and l thalamus) and CAF<br>- $_{-}$corr between FC (l rPFC and mPFC, r thalamus and DMN and l/r PPC, l thalamus and PCC) and CAF
Franciotti et al. (2013) [44]	18 (9:9)<br>75.0 ± 1.0<br>MMSE:<br>20.6 ± 0.5<br>Diagnosis criteria:<br>McKeith et al., 1996<br>Medication:<br>0 patients on ChEI.	15 (10:5)<br>74.0 ± 2.0<br>MMSE:<br>28.9 ± 0.8	AD	Clinical assessment	1.5 Philips	9 ROIs (1000 voxels per ROI centred on DMN clusters), ROI-to-ROI Pearson product-moment analysis, Granger causality analysis	- interhemispheric connectivity in frontal and parietal areas ↓<br>- connectivity between frontal and parietal areas in r hemisphere ↓<br>- no interhemispheric causal connection in DLB	- $_{-}$corr: FC values in r middle frontal gyrus and r lateral parietal cortex and CAF<br>- impairment of connection between r frontal and parietal regions associated with flCog<br>- Loss of information on directionality in frontoparietal connections in r hemisphere in low fluctuators

Galvin et al. (2011) [43]	15 (4:11)<br>71.7 ± 9.1<br>MMSE:<br>25.0 ± 4.4<br>Diagnosis criteria:<br>McKeith et al., 2005<br>Medication:<br>All patients on ChEI, 6 on dopaminergic medication.	38 (26:12)<br>73.9 ± 6.6<br>MMSE:<br>28.8 ± 1.2	AD		3.0 T<br>Siemens Allegra	Seed-to-voxel<br>Pearson product-moment correlation<br>Seed region: combined bilateral precuneus (±7, −60, 21)	- FC ↓ between precuneus and mPFC and secondary visual cortex<br>- correlations between precuneus and primary visual cortex and l/r hippocampus changed from + to −<br>- −corr between precuneus and rostral ACC and protoparietal operculum increased<br>- −corr between precuneus and superior frontal gyrus, putamen and IPS to +corr in DLB	
Kenny et al. (2012) [46]	15 (N/P)<br>80.6 ± 6.0<br>MMSE:<br>19.5 ± 4.2<br>Diagnosis criteria:<br>McKeith et al., 1996; 2005<br>Medication:<br>10 patients on ChEI, 6 on antidepressants, 1 on non-benzodiazepine.	16 (N/P)<br>76.3 ± 8.3<br>MMSE:<br>28.6 ± 1.3	AD		3.0 T<br>Philips Intera Achieva	Seed-to-voxel<br>Seed regions: l/r hippocampi, l/r PCC, precuneus, visual cortices	- FC ↑ between r PCC and l ACC, r globus pallidus, anterior lobe and posterior lobe	
Kenny et al. (2013) [47]	15 (6:9)<br>80.6 ± 6.0<br>MMSE:<br>19.5 ± 4.2<br>Diagnosis criteria:<br>McKeith et al., 1996; 2005	16 (7:9)<br>76.3 ± 8.3<br>MMSE:<br>28.6 ± 1.3	AD		3.0 T<br>Philips Intera Achieva, 8 channel head coil	Seed-to-voxel<br>Seed regions: l/r head of caudate nucleus, l/r putamen, l/r thalamus	- abnormal connectivity between l caudate and l PHG, r PCC and bilateral precuneus<br>- abnormal connectivity between r caudate and bilateral PCC, l precuneus, r culmen<br>- abnormal connectivity between l putamen and l pre-/postcentral	

	Medication:<br>10 patients on ChEI, 6 on antidepressants, 1 on non-benzodiazepine.						gyrus and l inferior parietal regions<br>- abnormal connectivity between l thalamus with bilateral cingulate, r insula and bilateral frontal regions<br>- abnormal connectivity between r thalamus with r frontal and r limbic regions	
Lowther et al. (2014) [45]	15 (6:9)<br>80.6 ± 6.0<br>MMSE:<br>19.5 ± 4.2<br>Diagnosis criteria:<br>McKeith et al., 1996; 2005<br>Medication:<br>Not medication-free but not specified.	40 (20:20)<br>77.8 ± 4.5<br>MMSE:<br>29.1 ± 1.2	AD	Clinical assessments	3.0 T Philips Intera Achieva, 8 channel head coil	ICA: 25 independent components relating to DMN, SA, ECN, BGN, LN	- FC ↓ in precuneus, inferior parietal, middle frontal, inferior temporal, lingual gyrus and posterior lobe of cerebellum (DMN)<br>- FC ↓ in frontal, occipital and parietal cortex (SA and ECN)<br>- FC ↑ in superior temporal regions (DMN)<br>- FC ↑ in frontal and limbic regions (BGN and LN)	- +corr between z-statistic score (in l superior frontal and l ACC) and UPDRS and CAF
Ma et al. (2019) [54]	17 (4:13)<br>71.9 ± 5.7<br>MMSE:<br>19.1 ± 4.8<br>Diagnosis criteria:<br>McKeith et al., 2005<br>Medication:<br>N/P.	20 (11:9)<br>67.8 ± 7.2<br>MMSE:<br>29.1 ± 0.9			3.0 T Siemens Verio, 8 channel head coil	236 ROIs (diameter: 5 mm) in 12 brain networks, DFC with Pearson's correlation, k-means clustering Graph-theoretical analysis	- DFC variations ↑ within networks (DMN, SMN, VN, FPN, SA, DAN, subcortical network)<br>- DFC variations ↓ within networks (DMN and SC) and between-networks (DMN, VN, and FPN and SMN and FPN)<br>- dwell time in state 2 ↓<br>- local efficiency ↓	
Peraza et al. (2014) [38]	16 (3:13)<br>76.2 ± 5.7<br>MMSE:<br>24.2 ± 3.8	17 (3:14)<br>77.3 ± 4.7<br>MMSE:		Clinical assessments	3.0 T Philips Intera Achieva	11 resting state networks identified by MELODIC: central and lateral	- FC ↓ in l FPN (l pallidum, l/r putamen, lingual gyrus, intracalcarine cortices and r frontal operculum), temporal	- corr for l FPN and CAF

	Diagnosis criteria: McKeith et al., 2005<br>Medication: 13 patients on ChEI, 2 on benzodiazepine.	29.1 ± 0.8				VNs, DMN I and II, l/r FPN, SMNs, temporal networks	network (l/r lingual gyrus, r putamen, r precentral gyrus, l cingulate gyrus and l/r intracalcarine cortices) and SMNs (l/r lateral occipital cortex, l/r lingual gyrus, l supramarginal gyrus)	
Peraza et al. (2015) a [37]<br>*Neurobiology Aging*	18 (5:13)<br>77.2 ± 6.2<br>MMSE: 23.6 ± 3.9<br>Diagnosis criteria: McKeith et al., 2005<br>Medication: N/P.	17 (3:14)<br>76.8 ± 5.7<br>MMSE: 29.1 ± 0.9	AD		3.0 T Philips Intera Achieva	12 ROIs from ICA, graph-theoretical analysis	- correlation strength ↓ for middle and long distance ranges<br>- modularity ↑ (trend)<br>- node degree ↓ in parietal and posterior temporal cortices, and l hippocampus<br>- node degree ↑ in thalami<br>- nodal clustering coefficient ↓ in most of cortex<br>- betweenness centrality ↓ in temporal and parietal regions<br>- global efficiency ↑<br>- characteristic path length ↓<br>- normalized clustering coefficient ↑<br>- small-worldness ↑ (trend)	- +corr between *E* and MMSE
Peraza et al. (2015) b [39]<br>*Int J Geriatr Psychiatry*	18 (5:13)<br>77.2 ± 6.1<br>MMSE: 23.6 ± 3.9<br>Diagnosis criteria: McKeith et al., 2005<br>Medication: Some patients on ChEI.	17 (3:14)<br>76.9 ± 5.8<br>MMSE: 29.1 ± 0.9	PDD	Clinical assessments	3.0 T Philips Intera Achieva	Seed-to-voxel FC, 12 seeds in DMN (mPCC, mPFC, medial precuneus), FPN (l/r posterior IPS, l/r anterior IPS), and MN (l/r putamen, l/r thalamus, SMA)	- FC ↓ between DMN seed and cerebellar regions, intracalcarine cortices, occipital cortices, temporal cortices, and cingulate gyri<br>- FC ↓ between r precuneus and thalamus and pallidum<br>- FC ↓ for FPN seeds in motor-sensory cortices, pre-/postcentral gyri<br>- FC ↓ between SMA and postcentral gyri, lateral occipital	

							cortices, cingulate, precuneus and cerebellum	
Peraza et al., 2016 [48]	19 (6:13)<br>76.3 ± 6.5<br>MMSE:<br>23.1 ± 4.1<br>Diagnosis criteria:<br>McKeith et al., 2005 (presumably).<br>Medication:<br>10 patient on ChEI, 10 on L-DOPA.	16 (3:13)<br>76.8 ± 5.3<br>MMSE:<br>29.1 ± 0.9	AD	VBM, clinical assessments	3.0 T Philips Intera Achieva	ReHo, seeds in cuneus, precuneus, sensory-motor cortices, thalamus, putamen	- ReHo values ↓ in sensory-motor areas, esp. in l/r precentral gyri<br>- ReHo values ↑ in l MTG and r central opercular cortex	- +corr between r l/r thalamus ReHo and MMSE<br>- +corr between l caudate and left putamen and UPDRS<br>- +corr between r putamen and CAF<br>- +corr between l/r cuneus and VHs
Schumacher et al. (2018) [36]	31 (12:19)<br>78.1 ± 6.7<br>MMSE:<br>22.0 ± 4.3<br>Diagnosis criteria:<br>McKeith et al., 2005<br>Medication:<br>28 patients on ChEI, 18 on anti-parkinson medication.	31 (9:22)<br>76.4 ± 7.2<br>MMSE:<br>28.9 ± 1.1	AD	Clinical assessments	3.0 T Philips Intera Achieva	FC between 27 networks identified with ICA in independent cohort	- FC ↓ within lateral and medial SMN, temporal network, BGN, r MN, thalamic network, insular network 1, anterior cingulate network, temporal pole network<br>- FC ↑ within l MN, VAN, insular network<br>- FC from negative (in HC) to around zero in temporal pole to anterior cingulate networks (in DLB)	- n.s. corr with clinical scores after correction for multiple comparisons
Schumacher et al. (2019) [53]	31 (12:19)<br>78.1 ± 6.7<br>MMSE:<br>22.0 ± 4.3<br>Diagnosis criteria:	31 (9:22)<br>76.4 ± 7.2<br>MMSE:<br>28.9 ± 1.1	AD	Clinical assessments	3.0 T Philips Intera Achieva	27 ICA RSNs, ROI-to-ROI covariance matrices, k-clustering, dynamic network analysis,	Dynamic connectivity<br>- n.s.<br>k-meanes clustering<br>- number of state transitions and intertransitional intervals n.s.<br>- state 1 frequency and dwell time ↓	- n.s. corr between frequency of state 2 with UPDRS (DLB) after correction

	McKeith et al., 2005<br>Medication:<br>28 patients on ChEI, 18 on anti-parkinson medication.					graph-theoretical analysis	- state 2 frequency and dwell time ↑<br>Dynamic network measures<br>- local efficiency variability n.s.<br>- global efficiency variability ↓	for multiple correlation
Schumacher et al. (2021) a [55]<br>*Neurology*	LB-MCI<br>31 (2:29)<br>74.7 ± 6.6<br>MMSE:<br>26.6 ± 2.6<br>Diagnosis criteria:<br>McKeith et al., 2017<br>Medication:<br>14 patients on ChEI, 2 on anti-parkinson medication.	24 (7:17)<br>73.5 ± 7.6<br>MMSE:<br>28.3 ± 1.1	AD-MCI		3.0 T Philips Intera Achieva	21 and 51 resting state networks defined with low- and high-dimensional ICA, dynamic sliding window analysis + leading eigenvector dynamic analysis	Static functional connectivity<br>- n.s.<br><br>Dynamic sliding window analysis<br>- number of state transitions, mean intertransition time, frequency of the 3 states, or mean dwell time per state n.s.<br><br>Leading eigenvector dynamic analysis<br>- number of state transitions, mean intertransition time, frequency of the 3 states, or mean dwell time per state n.s.<br>- no impact of ChEI intake on results	
Schumacher et al. (2021) b [49]<br>*Brain*	27 (3:24)<br>69.6 ± 7.1<br>MMSE: N/P<br>Diagnosis criteria:<br>McKeith et al., 2005; 2017<br>Medication:<br>N/P.	99 (7:92)<br>69.0 ± 10.3<br>MMSE:<br>N/P		PiB-PET, flortaucipir-PET	3.0 T GE	Posterior DMN connectivity and ROI-to-ROI connectivity between 100 cortical ROIs, Pearson's correlations	- posterior DMN connectivity: n.s.	- posterior DMN connectivity ~ overall mean flortaucipir SUVr and cortical PiB SUVr<br>- +corr between FC and tau covariance<br>- +corr between FC seed-to-target region and flortaucipir

								in seed/target region
Sourty et al. (2016) [42]	16 (8:8)<br>74.7 [54-89]<br>MMSE:<br>20.8 ± 3.2<br>Diagnosis criteria:<br>McKeith et al., 2005<br>Medication:<br>N/P.	20 (11:9)<br>64.4 [46-76]<br>MMSE:<br>29.0 ± 1.0			3.0 T Siemens Verio	DFC in 7 RSNs (DMN, l/r FPN, OPFN, OPN, MON, BGN), Product hidden Markov models	- probability ↓ for MON and r FPN in "correlated" state<br>- more "correlated" l FPN and more "anti-correlated" r FPN (mean occupation rate 40 % in DLB and 22% in HC)<br>All participants:<br>- states with highest probability with OPN, OPFN, and/or MON in "correlated" state	

1) Total number of participants. Ratio between females and males. Mean ± standard deviation (SD) of age and MMSE are reported as available. If only median ± standard error or range are available, these numbers are provided in brackets.
2) Results refer to comparison of DLB patients to HCs if not specified otherwise.

ACC = anterior cingulate cortex. AD = Alzheimer's disease. AD-MCI = Mild cognitive impairment with Alzheimer's disease. BGN = basal ganglia network. CAF = Clinician Assessment of Fluctuation. ChEI = acetylcholinesterase inhibitor. +corr = positive correlation. -corr = negative correlation. DAN = dorsal attention network. DFC = dynamic functional connectivity. DLB = dementia with Lewy bodies. FC = functional connectivity. flCog = fluctuating cognition. FPN = frontoparietal network. DMN = default mode network. ECN = executive control network. FEF = frontal eye field. fMRI = functional magnetic resonance imaging. HC = healthy control. ICA = independent component analysis. IPS = intraparietal sulcus. l = left. LB-MCI = Mild cognitive impairment with Lewy bodies. LN = limbic network. lPFC =lateral prefrontal cortex. MMSE = Mini Mental State Examination. MN = motor network. MON = medial occipital network. mPFC = medial prefrontal cortex. mPCC = middle posterior cingulate cortex. MTG = middle temporal gyrus. N/P = not provided. n.s. = not significant. OPFN = occipito-parieto-frontal network. OPN = occipital posterior network. PCC = posterior cingulate cortex. PD = Parkinson's disease. PDD = Parkinson's disease dementia. PET = photon emission tomography. PHG = parahippocampal gyrus. PiB-PET = ^{11}C Pittsbourgh compound B photon emission tomography. r = right. ReHo = regional homogeneity. ROI = region of interest. rPFC = rostral prefrontal cortex. RSN = resting state network. SA = salience network. SMA = supplementary motor area. SMN = sensorimotor network. SUVr = standardized uptake value ratio. UPDRS = Unified Parkinson's Disease Rating Scale. VAN = ventral attention network. VBM = voxel-based morphometry. VH = visual hallucination. VN = visual network.

S7 ^{18}FDG-PET-based connectivity in DLB.

Authors (year)	DLB patients (F:M) age MMSE Diagnosis criteria Medication [1)]	HC (F:M) age MMSE [1)]	Other patient groups	Additional measures	Acquisition Details	Methods	Connectivity results [2)]	Correlations with other measures
Caminiti et al. (2017) [56]	42 (15:27)<br>72.3 ± 6.7<br>MMSE : N/P<br>Diagnosis criteria: McKeith et al., 2005<br>Medication: N/P.	42 (20:22)<br>72.1 ± 5.9<br>MMSE : N/P			Discovery STE PET, GE	121 ROIs (cortical, subcortical, cerebellar and brainstem structures), further division in 12 submatrices, analysis for α-synuclein, dopaminergic and cholinergic neurotransmission systems, graph-theoretical analysis	Whole brain connectivity<br>- clustering coefficient ↓<br>- modularity ↓ (6 modules in HC and 8 modules in DLB)<br>- global efficiency ↑<br>- characteristic path length ↓<br>- number of long-distance connections ↑<br>- number of short-distance connections ↓<br>- number of hubs (21 in HC and 9 in DLB, lost hubs in frontal, parietal, occipital, thalamic and cerebellar regions)<br>Submatrix connectivity<br>- local connectivity ↓ in occipital cortex, cerebellum, thalamus, and brainstem<br>- local connectivity ↑ in parietal, temporal, frontal matrices, and basal ganglia<br>- long distance connections ↓ in frontal with occipital and cerebellar regions, in thalamus with occipital	

							cortex, median cingulum, paracentral lobule, cerebellum and brainstem, in cerebellum to frontal, occipital, thalamic, and brainstem submatrices<br>- long distance connections ↑ in frontal to parietal and basal ganglia matrices<br>A-synuclein spreading<br>- disconnection from brainstem to hippocampus and amygdala, gradient from brainstem outwards<br>Dopaminergic network<br>- loss of connections from dorsal striatum to prefrontal, sensorimotor and supplementary motor region while mesolimbic network connectivity was more spared<br>Cholinergic networks<br>- number of connections ↓ in Ch1-Ch2 (especially hypothalamic regions), Ch5-Ch6<br>- number of connections ↑ in Ch3 and lateral Ch4<br>- mixed profile in medial and lateral capsular Ch4 nuclei pathways	
Carli et al. (2020) [12]	30 (8:22)<br>74.0 ± 6.6<br>MMSE: 19.5 ± 5.83<br>Diagnosis criteria: N/P	50 (22:28)<br>68.4 ± 9.0<br>MMSE : N/P	PD, RBD		Discovery STE GE PET	nigro-striato-cortical dopaminergic, noradrenergic and cholinergic networks	- alterations in nigro-striato-cortical dopaminergic network connectivity in 11 out of 18 ROIs (bilateral caudate nuclei, r putamen, bilateral globus pallidus, r motor section of thalamus,	

	Medication: N/P but potentially dopaminergic medication for some patients.						r MFG, l IFG pars opercularis, r SFG, bilateral pre- and postcentral gyri)<br>- complete reconfiguration of noradrenergic network (26 out of 26 ROIs affected)<br>- CH3, medial Ch4, and Ch5-Ch6 division networks extensively reconfigured	
Chen et al. (2018) [10]	22 (1:21)<br>66.9 ± 8.4<br>MMSE: 20.0 ± 5.0<br>Diagnosis criteria: N/P<br>Medication: N/P.	22 (17:5)<br>63.5 ± 5.6<br>MMSE: 28.9 ± 1.3			Siemens Biograph 64 PET/CT	90 ROIs, covariance correlation matrix, hubs (betweenness centrality > 1.5) used as seed regions: seed-to-voxel, graph-theoretical analysis	Network parameters<br>- altered clustering topography in covariance matrix<br>- normalized clustering coefficient ↓<br>- small-worldness ↓<br>- normalized characteristic path length ↑<br>- differences in clustering coefficient, characteristic path length, global and local efficiency<br>Hub regions<br>- 16 hubs in HC (in prefrontal and occipital cortices) and 21 hubs in DLB (in prefrontal, occipital, and subcortical regions)<br>- betweenness centrality ↑ in r olfactory area, r hippocampus, and l fusiform gyrus<br>- betweenness centrality ↓ in r IFG<br>Seed correlation (from r IFG)	

							- correlation ↑ to frontal regions<br>- correlation ↓ to occipital regions	
Chen et al. (2019) [57]	22 (1:21)<br>66.9 ± 8.4<br>MMSE: 20.0 ± 5.0<br>Diagnosis criteria: McKeith et al., 2005<br>Medication: N/P.	22 (17:5)<br>63.5 ± 5.6<br>MMSE: 28.9 ± 1.3	AD, PDD		Siemens Biograph 64 PET/CT	90 ROIs, ROI-to-ROI correlation, graph-theoretical analysis	Network parameters<br>- small-worldness ↓<br>- clustering coefficient ↑<br>- characteristic path length ↑<br>- global and local efficiency ↓<br>Hub regions<br>- 15 hubs in HC (prefrontal and occipital cortex), and 20 hubs in DLB (in prefrontal, occipital, and subcortical regions)<br>Seed correlation (from r MTG)<br>- correlation ↑ to MFG and precuneus (in DLB)<br>- correlation ↓ to fusiform and parietal gyrus (in DLB)<br>Asymmetry of network efficiencies<br>- global and local efficiency: rightward asymmetry in DLB	
Huber et al. (2020) [13]	84 (37:47)<br>72.6 ± 7.0<br>MMSE: 22.6 ± 4.3<br>Diagnosis criteria: N/P<br>Medication: N/P.	28 (11:17)<br>73.2 ± 7.6<br>MMSE: N/P		DaT-SPECT		Pearson’s correlation coefficient for biomarkers in putamen<br>Voxel-wise regression analysis with putamen DaT Z-scores as predictor and FDG-PET SUVr	Metabolic connectivity at different stages<br>- strongest alterations in basal ganglia and limbic system: connectivity ↑ in mild dopamine deficiency but connectivity ↓ in moderate and severe dopamine deficiency<br>- metabolic connectivity ↓ between parieto-occipital areas and limbic system/basal ganglia,	- -corr between DaT Z-scores and FDG-PET SUVr in putamen<br>Voxel-wise regression<br>- -corr between DaT Z-scores and FDG-PET SUVr in basal ganglia and limbic system (putamen,

						as outcome variable, Metabolic connectivity from inter-regional coefficients of FDG-PET SUVr (77 ROIs) in 3 different stages of dopamine defficiency	enhanced with greater dopamine deficiency<br>- metabolic connectivity ↓ for regions with higher association between dopamine deficiency and relative glucose metabolism<br>➔ linkage between relative hypermetabolism associated with dopaminergic loss and decreased metabolic connectivity at regional level	thalamus, ACC, PCC)<br>- +corr in parietal and occipital cortices and cerebellum<br>ROI-based linear regression<br>- −corr in r NAcc, r thalamus, l/r ACC, r PCC, r subcallosal area
Iaccarino et al. (2018) [11]	38 (13:25)<br>72.9 ± 7.5<br>MMSE: 16.9 ± 4.7<br>Diagnosis criteria: N/P<br>Medication: Patients on range of medication but not specified.	38 (18:20)<br>71.5 ± 6.9<br>MMSE: N/P		Clinical assessments	General Electric Discovery STE PET/CT	Correlation between voxel-wise FDG-PET uptake and regional average uptake in seeds, Seed regions: VNs (calcarine and lateral occipital cortex), VAN/DAN (anterior insula, IPL), DMN (PCC, precuneus)	Within-network analyses:<br>- network extent ↓ (variation most notable in primary VN and VAN in DLB-NVH and DMN, DAN, higher VN in DLB-VH)<br><br>Between-network analyses:<br>- connectivity loss between DAN and VAN (significantly associated in HC)	- −corr between brain hypometabolism in r occipital-temporal cluster and VHs
Imai et al. (2020) [58]	18 (6:12)<br>77.0 ± 7.0<br>MMSE: 23.0 ± 4.0<br>Diagnosis criteria: McKeith et al., 2017	142 (127:15)<br>67.0 ± 5.0<br>MMSE: 29.0 ± 1.0	AD		Headtome-V/SET 2400W Scanner	Pearson correlation between 68 cortical ROIs (34 per hemisphere), graph-theoretical analysis	- more heterogeneous correlation matrix<br>Global parameters<br>- average strength ↓<br>- global efficiency ↓<br>- local efficiency ↓<br>- clustering coefficient ↓<br>- transitivity ↓<br>- average eccentricity ↑	

	Medication:<br>N/P.						- average characteristic path length ↑<br>- modularity ↑	
Sala et al. (2019) [59]	DLB<br>72 (30:42)<br>72.4 ± 7.6<br>MMSE:<br>18.7 ± 4.5<br>Diagnosis criteria:<br>McKeith et al., 2005<br>Medication:<br>N/P.	93<br>(46:47)<br>69.7 ±<br>6.05<br>MMSE :<br>N/P		Clinical and neuropsychological assessments	Discovery STE PET	ROI-to-ROI, Pearson's product momentum coefficients,<br>Seed regions:<br>DMN (ACC/vmPFC, PCC/precuneus), VNs (calcarine and lateral occipital cortex), SA/DAN (anterior insula, IPL), EN (dlPFC)	- changes in between-network interactions converge on visual and attention networks, not only reduction but reversal of correlation	- VHs connected to changes in SA, DAN, VNs<br>- RBD connected to changes in SA, DAN, LN<br>- +corr between VN and Attentive Matrices score<br>- +corr between EN and Raven's Colored Progressive Matrices score

1) Total number of participants. Ratio between females and males. Mean ± standard deviation (SD) of age and MMSE are reported as available..
2) Results refer to comparison of DLB patients to HCs if not specified otherwise.

ACC = anterior cingulate cortex. AD = Alzheimer's disease. ChEI = acetylcholinesterase inhibitor. +corr = positive correlation. -corr = negative correlation. DAN = dorsal attention network. DaT-SPECT = ^{123}I-Ioflupane brain single-photon emission computed tomography. DLB = dementia with Lewy bodies. dlPFC = dorsolateral prefrontal cortex. DMN = default mode network. EN = executive network. FDG-PET = ^{18F}2-fluoro-deoxy-d-glucose photon emission tomography. HC = healthy control. IFG = inferior frontal gyrus. IPL = inferior parietal lobule. L = left. LN = limbic network. MFG = middle frontal gyrus. MMSE = Mini Mental State Examination. MTG = middle temporal gyrus. N/P = not provided. Nacc = nucleus accumbens. NVH = no visual hallucination. PCC = posterior cingulate cortex. PD = Parkinson's disease. PDD = Parkinson's disease dementia. PET = positron emission tomography. R = right. RBD = REM-sleep behavioural disorder. ROI = region of interest. SA = salience network. STG = superior temporal gyrus. SUVr = standardized uptake value ratio. VAN = ventral attention network. VH = visual hallucination. vmPFC = ventromedial prefrontal cortex. VN = visual network.

S8 SPECT-based connectivity in DLB.

**Authors (year)**	**DLB patients (F:M) age MMSE Diagnosis criteria Medication**[1)]	**HC (F:M) age MMSE** [1)]	**Additional measures**	**Acquisition Details**	**Methods**	**Connectivity results** [2)]	**Correlations with other measures**
Colloby et al. (2020) [60]	14 (7:7)<br>74.1 ± 7.1<br>MMSE:<br>15.7 ± 6.2<br>Diagnosis criteria:<br>McKeith et al., 2017<br>Medication:<br>No antipsychotic, cholinergic, anticholinergic, antidepressant medications..	24 (9:15)<br>74.1 ± 5.1<br>MMSE:<br>28.3 ± 1.5	Cognitive/behavioural tests	QNB SPECT (M_1 and M_4 receptors)	Voxel-to-voxel covariance	- concomitant bilateral preserved/increased M_1/M_4 binding in medial/middle frontal gyrus, precuneus, cuneus and lingual gyrus with concomitant decreased binding in r MTG/STG, insula, l caudate regions	- correlations with cognitive/behavioural tests did not survive correction for multiple comparisons

1) Total number of participants. Ratio between females and males. Mean ± standard deviation (SD) of age and MMSE are reported as available. If only median ± standard error or range are available, these numbers are provided in brackets.
2) Results refer to comparison of DLB patients to HCs if not specified otherwise.

DLB = dementia with Lewy bodies. MMSE = Mini Mental State Examination. l = left. MTG = middle temporal gyrus. QNB = ^{125}I-iodo-quinuclidinyl-benzilate. r = right. STG = superior temporal gyrus.

S9 EEG-based connectivity in DLB.

**Authors (year)**	**DLB patients (F:M) age MMSE Diagnosis criteria Medication** [1)]	**Controls (F:M) age MMSE** [1)]	**Other patient groups**	**Additional measures** [2)]	**Acquisition Details**	**Methods** [3)]	**Connectivity results** [4)]	**Correlations with other measures** [5)]
Andersson et al. (2008) [62]	20 (10:10)<br>[77, 54-85]<br>MMSE:<br>22.0 ± 4.0<br>Diagnosis criteria:<br>McKeith et al., 1996<br>Medication:<br>No ChEI before EEG recording.	54 (34:20)<br>[72, 60-94]<br>MMSE:<br>29.0 ± 1.0	AD		Nervus system, 19 electrodes, 256 Hz sampling rate	Coherence for all electrode pairs, 4 frequency bands (delta, theta, alpha, beta)	- delta band coherence ↑<br>- alpha band coherence ↓	
Babiloni et al. (2018) [64]	34 (23:11)<br>75.1 [± 1.1]<br>MMSE:<br>18.6 [± 0.8]<br>Diagnosis criteria:<br>McKeith et al., 2005<br>Medication: 25 patients on ChEI, 17 on dopamine agonists	40 (24:16)<br>72.9 [± 1.1]<br>MMSE<br>28.7 [± 0.2]	AD, PDD		21 electrodes, ≥ 128 Hz sampling rate	LLC for 5 ROIs (frontal, central, parietal, occipital, temporal),<br>8 frequency bands (delta, theta, alpha 1 – 3, beta 1,2, gamma)	Interhemispheric LLC<br>- similar spatial and frequency profile of interhemispheric LLC (across patient groups)<br>- magnitude in alpha range ↓ in parietal, occipital, and temporal regions<br>Intrahemispheric LLC<br>- similar spatial and frequency profile of interhemispheric	- +corr between interhemispheric LLC solutions in temporal alpha 3 and MMSE<br>- +corr between intrahemispheric LLC solutions in central, parietal, occipital alpha 3 and MMSE (across all groups)

							LLC (across patient groups)<br>- magnitude in alpha range ↓ in central, parietal, occipital, and temporal regions<br>➔ differential solutions for patient groups (AD<DLB<PDD)<br><br>- ROC classification between DLB and HC based on inter- and intrahemispheric LLC solutions	
Babiloni et al. (2019) [65]	LB-MCI<br>23 (9:14)<br>75.7 [± 1.4]<br>MMSE: 25.7 [± 0.4]<br>Diagnosis criteria: McKeith et al., 2005; 2017<br>Medication: 24 – 48 h washout of medication before EEG recording.	30 (12:18)<br>74.7 [± 0.8]<br>MMSE: 28.5 [± 0.2]	AD-MCI		19 electrodes, ≥128 Hz sampling rate	LLC for 5 ROIs (frontal, central, parietal, occipital, temporal)<br>8 frequency bands (delta, theta, alpha 1,2,3, beta 1,2, gamma)	Interhemispheric LLC<br>- magnitude of LLC solutions ↓ in all ROIs in alpha 2,3<br><br>Intrahemispheric LLC<br>- magnitude of LLC solutions ↓ in all ROIs in alpha 2,3<br><br>- ROC discrimination between HC and LB-MCI only on intrahemispheric LLC solutions in global alpha 2	- +corr between interhemispheric LLC in global alpha 2 and MMSE score (across all groups)<br>- +corr between intrahemispheric LLC in global alpha 3 and MMSE score (across all groups)

Dauwan et al. (2016) [69]	66 (14:52)<br>70.0 ± 9.0<br>MMSE: 23.0 ± 5.0 (n=59)<br>Diagnosis criteria: McKeith et al., 2005.<br>Medication: 16 patients on non-specified CNS medication.	66 (14:52)<br>70.0 ± 7.0<br>MMSE: 28.0 ± 1.0<br><br>Medication: 6 HCs on non-specified CNS medication.	AD	Neuropsychological assessments	Brainlab, 21 electrodes, 500 Hz sampling rate	Directed connectivity with PTE for all electrode pairs, 3 frequency bands (theta, alpha, higher beta)	- disturbance in posterior-to-anterior pattern of information flow with normalized PTE gradient shift from posterior to more anterior in alpha band<br>- differences in mean normalized PTE at electrodes P3 and T5	- corr between higher mean normalized PTE in posterior areas in beta bands and better performance in TMT B test
Kai et al. (2005) [61]	DLB+donepezil<br>7 (N/P)<br>76.0 ± 4.0<br>MMSE:<br>20.0 ± 5.0<br><br>DLB-donepezil<br>15 (N/P)<br>74.0 ± 6.0<br>MMSE:<br>19.0 ± 4.0<br><br>Diagnosis criteria: McKeith et al., 1996<br>Medication: Donepezil according to group.	12 (N/P)<br>72.0 ± 6.0<br>MMSE: N/P	AD+donepezil,<br>AD-donepezil		NEC Synafit 1000, 14 electrodes, 1000 Hz sampling rate	Coherence for all electrode pairs,<br>4 frequency bands (delta, theta, alpha, beta)	DLB-donezepil vs HC<br>- delta power ↑<br>- theta power ↑ (higher power compared to patients taking donepezil, no difference between patients taking donepezil and HC)<br>- interhemispheric coherence ↓ in delta band (F3–F4, P3–P4, T3–T4, and T5–T6), in theta band (F3–F4), in alpha band (F7–F8, F3–F4, C3–C4, P3–P4, at T5–T), in beta band (C3-C4)	

							- interhemispheric coherence ↑ in theta band (T3-T4)<br>- intrahemispheric coherence ↓ in delta, theta, alpha, and beta bands (45 electrode pairs)<br><br>DLB+/-donepezil<br>- no differences in interhemispheric coherence<br>- intrahemispheric coherence ↓ in delta band (F3-F7), beta-band (T3-T5), and delta band (F3-F7)	
Mehraram et al. (2020) [68]	25 (5:20)<br>76.2 ± 6.2<br>MMSE:<br>22.7 ± 4.3<br>Diagnosis criteria:<br>McKeith et al., 2005; 2017<br>Medication:<br>22 patients on ChEI.	18 (7:11)<br>76.3 ± 5.5<br>MMSE:<br>29.2 ± 0.9	AD, PDD	Cognitive and clinical assessments	Waveguard, 128 Ag/AgGl electrodes, 1024 Hz sampling rate,	Weighted PLI over 3 distance ranges, 3 frequency bands (theta, alpha, beta), graph-theoretical analysis	- average weighted PLI ↓ in alpha band in long connections and in beta band for all distance ranges<br>- parietal-frontal connectivity particularly affected<br>- occipital-central patterns ↓ in beta-band networks<br>Network properties	- -corr between average weigthed PLI in alpha and beta bands and VHs<br><br>- -corr between average clustering coefficient in alpha and animal naming test<br><br>- -corr between node degree

							- network segregation ↑ in theta band<br>- nodal measures ↓ in alpha band<br>- network integration ↓ (characteristic path length ↑ and modularity ↑<br>- clustering coefficient in beta band in frontal and posterior areas and in alpha band in frontal, central, and posterior areas<br><br>- ROC classification with all weighted network measures: 4 most important variables: weighted PLI in beta band, modularity, characteristic path length, and clustering coefficient in alpha band (DLB+PDD vs HC)	and verbal fluency<br><br>- +corr between average characteristic path length and verbal fluency

Peraza et al. (2018) [67]	25 (5:20)<br>75.8 ± 6.5<br>MMSE: 22.6 ± 4.3<br>Diagnosis criteria: McKeith et al., 2005; 2017<br>Medication: 23 patients on ChEI.	17 (7:10)<br>76.2 ± 5.7<br>MMSE: 29.1 ± 0.9	AD, PDD	Cognitive and clinical assessments	Waveguard, 128 Ag/AgGl electrodes, 1024 Hz sampling rate	PLI for all electrode pairs, 6 frequency bands (delta, theta, high-theta, alpha, beta, dominant frequency band), graph-theoretical analysis	- degree ↓ (esp. in alpha band)<br>- leaf ratio ↓ (esp. in alpha band)<br>- diameter, radius ↑ (esp. in high-theta band)<br>- eccentricity ↑ (esp. in high-theta band)<br>- ! none of them significant in post-hoc tests<br>- PLI measures (mean, leaf, root, height) ↓ in alpha band	- +corr between dominant frequency and verbal fluency, animal-naming, CAMCOG, MMSE, TMT across all dementia groups and within AD and DLB<br>- -corr between network mean PLI in high-theta and VHs
Pugnetti et al. (2010) [63]	10 (7:3)<br>75.7 ± 5.3<br>MMSE: 21.0 ± 3.0<br>Diagnosis criteria: McKeith et al., 1996<br>Medication: Some patients on non-specified medication. Patients taking benzodiazepines, antidepressants, and neuroleptics were excluded.	14 (8:6)<br>68.5 ± 3.9<br>MMSE: 27.7 ± 2.0	PDD, PD	EEG + 12-Hz intermittent photic stimulation	Electro-Cap International, 30 electrodes, 500 Hz sampling rate	Global field synchronization over 19 electrodes, 7 frequency bands (delta, theta, alpha1, alpha2, beta1, beta2, beta3)	- global field synchronization ↓ in alpha 1	

Van Dellen et al. (2015) [66]	66 (14:52)<br>70.0 ± 9.0<br>MMSE: 23.0 ± 5.0 (n=59)<br>Diagnosis criteria: McKeith et al., 2005<br>Medication: 16 patients on non-specified CNS medication.	66 (14:52)<br>70.0 ± 7.0<br>MMSE: 28.0 ± 1.0<br>Medication: 6 HCs on non-specified CNS medication.	AD	Cognitive assessments	Brainlab, 21 electrodes, 500 Hz sampling rate	PLI for all electrode pairs, 4 frequency bands (delta, theta, alpha, beta)<br>graph-theoretical analysis	- alpha connectivity strength ↓<br>- alpha band network similarity ↓<br>- betweenness centrality ↓<br>- degree ↓<br>➔ Indicating loss of nodes<br>- MST diameter ↑, eccentricity range ↑, leaf fraction ↓<br>➔ Indicating lower efficiency	All in alpha band<br>- $_{+}$corr between leaf fraction in alpha and MMSE<br>- $_{-}$corr between diameter, eccentricity range in alpha and MMSE<br>- $_{+}$corr between leaf fraction, tree hierarchy, connectivity strength in alpha and VAT scores<br>- $_{+}$corr between connectivity strength in alpha and TMT scores

1) Total number of participants. Ratio between females and males. Mean ± standard deviation (SD) of age and MMSE are reported as available. If only median ± standard error or range are available, these numbers are provided in brackets.
2) Results refer to comparison of DLB patients to HCs if not specified otherwise.

AD = Alzheimer's disease. AD-MCI = mild cognitive impairment with Alzheimer's disease. CAMCOG = Cambridge Cognition Examination. ChEI = acetylcholinesterase inhibitor. $_{+}$corr = positive correlation. -corr = negative correlation.DLB = dementia with Lewy bodies. EEG = electroencephalography. HC = healthy control. l = left. LB-MCI = mild cognitive impairment with Lewy bodies. LLC = lagged linear connectivity. MMSE = Mini Mental State Examination. MST = minimum spanning tree. N/P = not provided. n.s. = not significant. PDD = Parkinson's disease dementia. PD = Parkinson's disease. PLI = phase lag index. PTE = phase transfer entropy. r = right. ROC = receiver operator characteristic. ROI = region of interest. TMT = Trail Making Test. VAT = Visual Association Test. VH = visual hallucinations
